# Comparative Transcriptome Analysis of Two Contrasting Chinese Cabbage (*Brassica rapa* L.) Genotypes Reveals That Ion Homeostasis Is a Crucial Biological Pathway Involved in the Rapid Adaptive Response to Salt Stress

**DOI:** 10.3389/fpls.2021.683891

**Published:** 2021-06-14

**Authors:** Na Li, Zhihuan Zhang, Zijing Chen, Bili Cao, Kun Xu

**Affiliations:** ^1^College of Horticulture Science and Engineering, Shandong Agricultural University, Tai’an, China; ^2^Collaborative Innovation Center of Fruit & Vegetable Quality and Efficient Production in Shandong, Tai’an, China; ^3^Key Laboratory of Biology and Genetic Improvement of Horticultural Crops in Huanghuai Region, Ministry of Agriculture and Rural Affairs, Tai’an, China; ^4^State Key Laboratory of Crop Biology, Tai’an, China

**Keywords:** Chinese cabbage, salt stress, ion homeostasis, photosynthesis, osmotic regulation, transcriptomics

## Abstract

Salt is the most important limiting factor in plant yield and quality. Different Chinese cabbage cultivars appeared different salt tolerances, but there are few studies attempting to elucidate the mechanism underlying this phenomenon. In this study, 100 mmol L^–1^ NaCl was found to be the most suitable treatment concentration according to a sprouting bag test of 39 Chinese cabbage cultivars, and through comprehensive comparison and analysis, the relative values of fresh weight and electrolyte leakage in leaves proved to be convenient indicators for the identification of salt tolerance in Chinese cabbage. We analyzed the physiological responses of Qinghua45 (salt-tolerant) and Biyuchunhua (salt-sensitive) in terms of the growth indexes, ion homeostasis and Photosynthesis, the results indicated that Qinghua45 could ensure osmotic regulation, ion homeostasis and photosynthesis under salt stress. Next, we compared the transcriptome dynamics of the two cultivars. Overall, 2,859 differentially expressed genes (DEGs) were identified, and the number of DEGs in Qinghua45 was significantly less than that in Biyuchunhua. VDAC promoted the release of Ca^2+^, which indirectly promoted the transport of Na^+^ to vacuoles through the SOS_2_ pathway. Cation/H (+) antiporter 17 and V-H + -ATPase improve the exchange of Na^+^ and H^+^ and maintain Na^+^ in the vacuoles, thereby reducing the injury affected by salt stress. Increases in galactinol synthase and soluble protein synthesis helped relieve osmotic stress caused by salt, together, they regulated the Na^+^ content and chlorophyll biosynthesis of the plant and enabled the plant to adapt to salt stress over time.

## Introduction

Salinity is a major environmental stress that negatively affects the growth and productivity of plant worldwide. Salinization of arable land is increasing, and it is estimated that by 2050, 50% of the world’s arable land will be affected by salinity (Bartels and Sunkar, 2005). World crop production is facing a crucial threat because of different environmental stresses, and salinity alone reduces crop yield by more than twofold (Hasanuzzaman et al., 2013).

Salt affected soils contain excessive soluble salts and exchangeable sodium on the surface or in the rhizosphere, which is associated with two main challenges to plants: osmotic stress and ionic stress. osmotic stress can due to the excess solutes outside the roots that reduce the ability of plants to extract soil water, and ionic stress which is usually caused by excessive influx of Na^+^ into the plant and lead to imbalanced metabolism, chlorosis, impaired photosynthesis, nutrient imbalance and yield loss (Deinlein et al., 2014; Hasanuzzaman et al., 2014; Geng et al., 2020). Growth inhibition is the most common physiological response of plants to salt, when salt accumulates above a certain concentration, it stops the plants from absorbing water and results in osmotic stress (Wu et al., 2016).

The accumulation of Na^+^ and K^+^ can play a role independently or in combination with other mechanisms in maintaining and adjusting the osmotic balance (Bayuelo-Jiménez et al., 2003). Under salt stress, an increase in Na^+^ content causes metabolism disorders and leads to the malabsorption of K^+^, Ca^2+^, Mg^2+^ and other mineral ions in plant (Parvaiz and Satyawati, 2008). In the mesophyll apoplast of leaves, insufficient osmotic adjustment results in reduced net photosynthesis because of stomatal closure (Flowers et al., 2015). Low calcium uptake by tomato has been linked with decreased transpiration rate, and the decreased absorption of Mg^2+^ shows serious impact on the photosynthesis of plant (Noble, 2018).

In recent years, many studies have explored the mechanism that governing salt tolerance in plants at the molecular level. After sensing salt stress, the signal transduction pathway has been found to be activated, and a large number of defense response-related genes were induced, which are mainly divided into the following five categories: (1) signal transduction-related proteins, (2) transcription factors (TFs), (3) proteins related to osmotic regulation, (4) antioxidant proteins and (5) genes induced by other salt treatments (Jamil et al., 2011). Studies have shown that the basic function of voltage-dependent anion channels (VDACs) in the signaling pathway is to promote and regulate the flow of metabolites between the cytoplasm and mitochondrial membrane (Camara et al., 2017). Osmotic stress induces a series of responses at the molecular and cellular levels, and the main events are the increase in intracellular calmodulin-like protein and calmodulin signal transduction, they can promote appropriate cellular responses in an effort to mitigate potential damage (Xiong and Zhu, 2002). The expression level of the V-H^+^-ATPase gene in leaves and roots increased under salt stress (Zhao et al., 2009). Chlorophyll synthesis plays an important role in plant stress, and previous studies on Arabidopsis thaliana Fluorescent (Flu) mutants also suggested that glutamyl-tRNA reduction is a limiting step in chlorophyll biosynthesis (Goslings et al., 2004).

Chinese cabbage, a leafy vegetable with a 1,500-year history, is now widely cultivated in China and other countries (Liu et al., 2010). Chinese cabbage is a glycophyte, and salt stress disturbs its photosynthesis and hormonal regulation, and causes nutritional imbalance, ion toxicity and osmotic stresses thereby reduce the yield and quality (Munns and Tester, 2008). However, the adaptation mechanism of plants to saline is still not clear; thus, understanding the responses of Chinese cabbage to salt stress and the associated tolerance mechanisms would help the development of strategies in improving the performance of Chinese cabbage under salt stress.

In this study, we established a simple way to evaluate the salt tolerance of Chinese cabbage, and two valid commercial Chinese cabbage cultivars were selected: Qinghua45 (QH) for its high-salt tolerance and Biyuchunhua (BY) for its sensitivity to salt stress. To gain insight into the mechanisms by which the cultivars improved the tolerance of Chinese cabbages and how are the related genes differently expressed under salt stress, we addressed the question of whether and how the increased tolerance of Chinese cabbage was associated with the maintenance of photosynthetic capacity, ion homeostasis and osmotic regulation. We treated Chinese cabbages with 100 mmol L^–1^ NaCl for 20 days and tried to analyzed the DEGs related to salt stress, mainly to identify key genes that play major roles under salt stress and how were they cooperated with physiological functions when treated with salt, further revealing the molecular mechanism of the salt tolerance response for the follow-up studies.

## Materials and Methods

### Plant Materials and Experimental Design

The experiment was carried out in a solar greenhouse located at Shandong Agricultural University in Tai’an (36° 09′ N, 117° 09′ E) in eastern China. Thirty-nine Chinese cabbage cultivars were provided by Degao. A germination experiment and a whole growth stage experiment were carried out to study the appropriate NaCl concentration and convenient screening indicators for salt tolerance in 39 Chinese cabbage cultivars. Four treatments of 0 mmol L^–1^ NaCl, 50 mmol L^–1^ NaCl, 100 mmol L^–1^ NaCl, and 200 mmol L^–1^ NaCl were designed for the sprouting bag test, add the four treatments water to the sprouting bag and place the seeds into the bag to observe the growth of the 39 Chinese cabbage cultivars. Two treatments of 0 mmol L^–1^ NaCl (marked as: CK) and 100 mmol L^–1^ NaCl (marked as: ST) were established for the whole growth stage experiment. Firstly, we sprouted the 39 Chinese cabbage seeds and sowed them in nursery trays containing substrate with standard irrigation and fertilization, when the seedlings were in the two-leaf one-heart stage, transplanted them to pottery (280-mm diameter, 300-mm height) containing 9 kg of substrate composed of a sandy loam-soil/peat mixture (1:1, v/v). NaCl (final NaCl concentration, 100 mmol L^–1^) was used as the salt stress, and a full-strength nutrient solution was used as the control. Plants were watered once every 2 days to maintain vermiculite moisture at 70–80%. Each treatment had four biological replicates, and each replication included seven seedlings. Sampling was performed at the seedling stage, rosette stage, and heading stage to measure the physiological indicators. Based on the results of the above two experiments, further research on relevant indexes and transcriptome analysis was carried out with Qinghua45 (QH, a salt-tolerant material) and Biyuchunhua (BY, a salt-sensitive material), two treatments and growth conditions are consistent with the whole growth stage experiment, after treated for 20 days, three independent biological replicates for each tissue sample were harvested, and samples were frozen immediately in liquid nitrogen and stored at −80°C for physiological measurement and RNA extraction.

### Measuring Methods

#### Growth Parameters

Fresh weight was measured gravimetrically.

#### Salt Injury Index

Seedlings were assessed by visual observation. Plants were classified for salt tolerance according to the salt injury index as described by Zhu et al. (2008) with some adjustments: 0 (no injury); 1 (the leaves of the plants are yellow, wilting or slightly curled); 2 (1/2 of the leaves are yellow, wilting and shrunk, with chlorotic or salty spots); 3 (2/3 of the leaves are yellow, leaves are wilting and dry); and 4 (all the leaves are wilting or dead). The salt injury index was calculated using the equation: salt injury index SI (%) = Σ (representative series × number of plants)/(the highest number × total number of plants) × 100.

#### Chlorophyll Content

Samples of 0.2 g leaves plus 20 ml 95% ethanol were sealed for more than 36 h in the dark until the leaves became white. The wavelengths 665 and 649 nm were selected for pigment content determination because they reflect the absorbance of Chlorophyll *a* and Chlorophyll *b*, respectively. The following equations were used:

$$\left[\mathrm{Chl}a\right]=13.95A_{665}-6.88A_{649}$$

$$\left[\mathrm{Chl}b\right]=24.96A_{649}-7.32A_{665}$$

$$\begin{array}{c} \mathrm{Chlorophyll}\mathrm{content}\left(\text{mg}/g\mathrm{FW}\right)\\ =\text{C}*\text{V}*\text{n}/\text{W}\left(V=0.02\text{L},n=1,W=0.2\text{g}\right) \end{array}$$

#### Leaf Area

The fifth and sixth leaves, which were counted from the bottom, were chosen and measured with a Licor leaf area meter LI-3100C.

#### Electrolyte Leakage

Seven discs of fresh leaves (1 cm diameter) were cut from the fully expanded leaves (five plants per treatment), and the samples were washed three times with deionized water to remove surface-adhered electrolytes. Leaf discs were placed in closed tubes containing 10 mL of deionized water and allowed to stand in the dark for 24 h at room temperature. The EC (EC1) of the bathing solution was determined at the end of the incubation period using a conduct meter LEICI-DDB-303A. The samples were then incubated in a water bath at 100°C for 20 min to release all electrolytes and cooled to 25°C, and their final electrical conductivity (EC2) was measured. EC (EC0) was the electrolyte content of deionized water. The electrolyte leakage (EL) was calculated as EL (%) = [(EC1 - EC0)/(EC2 - EC0)] × 100.

#### Na^+^ and K^+^ Content

The leaves were dried for 72 h at 75°C and ground separately in a Wiley mill to pass through a 20-mesh screen. Then, 0.5 g of the dried plant tissues was analyzed for Na^+^ and K^+^. They were determined by dry ashing at 400°C for 24 h, dissolving the ash in 1:20 HNO_3_, and assaying the solution obtained using an inductively coupled plasma emission spectrometer (iCAP 7000 SERIES; Thermo Fisher Scientific).

#### Relative Value

Relative values were calculated as the absolute value under 100 mmol L^–1^ NaCl stress treatment conditions/absolute value without NaCl addition.

#### Soluble Protein

The extraction and determination of soluble proteins was done separately for leaves. Samples [0.5 g of fresh weight (F.W.)] were homogenized in a chilled mortar with liquid nitrogen and dissolved in 50 mmol L^–1^ Na-PB pH 7.8 containing 1 mmol L^–1^ EDTA, PVPP, and protease inhibitor cocktail tablet (Roche complete). The solution was centrifuged at 10,000 × *g* at 4°C for 20 min and the supernatant was collected. Total soluble protein content was measured using bovine serum albumin (BSA) as a standard via the specific reaction of Coomassie Brilliant Blue G-250 dye with maximum absorbance at 595 nm.

#### The Photosynthetic and Chlorophyll Fluorescence Parameters

These were measured at 20 days after salt treatment and selected the seventh of the full-expansion function leaves from bottom to top. Photosynthetic parameters in fully expanded leaves, including Pn, WUE, GS, and Tr were determined within the time period of 8:30 am to 10:30 am using a CIRAS-3 Portable Photosynthesis System (CIRAS-3; PP Systems, United States).

#### Transcriptome Analysis

Transcriptome analysis was performed in Biomarker Technologies Corporation (Beijing, China). Leaves coming from three individual plants at the rosette stage were used for RNA isolation. Three biological replicates for each genotype and treatment, resulting in total 12 samples.

After the collection and preparation of samples, RNA quantification and qualification were carried out, and the clustering of the index-coded samples was performed on a cBot Cluster Generation System using TruSeq PE Cluster Kit v4-cBot-HS (Illumia) according to the manufacturer’s instructions. After cluster generation, the library preparations were sequenced on an Illumina platform and paired-end reads were generated. Subsequently, data analysis was performed, such as quality control, comparative analysis, gene functional annotation, SNP calling, quantification of gene expression levels, differential expression analysis, GO enrichment analysis, KEGG pathway enrichment analysis.

##### Quality control

The quality control was conducted according to the method of Zhang et al. (2019). All the downstream analyses were based on clean data with high quality.

Comparative analysis: The adaptor sequences and low-quality sequence reads were removed from the data sets. Raw sequences were transformed into clean reads after data processing. These clean reads were then mapped to the reference genome sequence. Only reads with a perfect match or one mismatch were further analyzed and annotated based on the reference genome. Hisat2 tools soft were used to map with reference genome.

##### Gene functional annotation

Gene function was annotated based on the following databases: Nr (NCBI non-redundant protein sequences); Nt (NCBI non-redundant nucleotide sequences); Pfam (Protein family); KOG/COG (Clusters of Orthologous Groups of proteins); Swiss-Prot (A manually annotated and reviewed protein sequence database); KO (KEGG Ortholog database); GO (Gene Ontology).

##### SNP calling

Picard—tools v1.41 and samtools v0.1.18 were used to sort, remove duplicated reads and merge the bam alignment results of each sample. GATK2 or Samtools software was used to perform SNP calling. Raw vcffiles were filtered with GATK standard filter method and other parameters (cluster Window Size: 10; MQ0 = 4 and [MQ0/(1.0^∗^DP)] > 0.1; QUAL < 10; QUAL < 30.0 or QD < 5.0 or HRun > 5), and only SNPs with distance > 5 were retained.

Quantification of gene expression levels: Gene expression levels were estimated by fragments per kilobase of transcript per million fragments mapped (FPKM).

Differential expression analysis: Differential expression analysis of two samples was performed using the EBseq. The FDR < 0.01 and | log2 (fold change) | ≥ 2 was set as the threshold for significantly differential expression.

##### GO enrichment analysis

Gene Ontology (GO) enrichment analysis of the differentially expressed genes (DEGs)was implemented by the GOseq R packages based Wallenius non-central hyper-geometric distribution (Young et al., 2010), which can adjust for gene length bias in DEGs.

##### KEGG pathway enrichment analysis

KEGG is a database resource for understanding high-level functions (Kanehisa et al., 2008) and utilities of the biological system, such as the cell, the organism and the ecosystem, from molecular-level information, especially large-scale molecular datasets generated by genome sequencing and other high-throughput experimental technologies^1^. We used KOBAS (Mao et al., 2005) software to test the statistical enrichment of differential expression genes in KEGG pathways.

#### Verification of Selected DEGs by RT-qPCR

Qinghua45 and Biyuchunhua were used as test materials and cultured under normal growth conditions. When growing to 1 week of seedling age, the Chinese cabbage seedlings were treated with 0 mmol L^–1^ NaCl and 100 mmol L^–1^, respectively. After 20 days, the third to fourth tender inner leaves were taken for storage in liquid nitrogen. According to transcriptome analysis, four candidate genes were selected, and the corresponding nucleotide sequences were searched from the transcriptome, and the selected DEGs were verified by RT-qPCR. The RT-qPCR primers designed for the selected DEGs genes are listed in Supplementary Table 5, BrActin gene was used as internal control gene. Total RNA was extracted from leaves of 3 independent biological replicates (0 mM NaCl and 100 mmol L^–1^ NaCl treatment) using the EASYspin RN09 RNA isolation kitSuper Mix (Aidlab, China) and cDNA synthesized using the HiScript II Q RT SuperMix (Vazyme, China). Real-time quantification was performed using 96-well plate real-time PCR system (CFX96 BIORAD), with the following steps: 95°C for 30 s for Prevalence, 40 cycles of 95°C for 10 s and 60°C for 30 s for the melting curve, 95°C for 15 s, 60°C for 60 s, and 95°C for 15 s. The qRT-PCR was amplified for 3 replications. The relative expression was calculated by 2^–Δ^ ^Δ^ ^*CT*^method (Livak and Schmittgen, 2001).

### Statistical Analysis

The plant sampling in this study followed the principle of random sampling. The data are presented as the mean of three replications and corresponding standard errors. All data were statistically analyzed by ANOVA using the DPS software package (DPS for Windows, 2009). Bar chart were used origin 9.1 software. The differences between the samples were determined by Duncan’s multiple range test at *P* < 0.05.

## Results

### Evaluation of Salt Tolerance of Different Chinese Cabbage Cultivars

To screen the appropriate concentration of NaCl, we carried out a sprouting bag test on 39 Chinese cabbage cultivars (Supplementary Figure 1). The length of roots were recorded in Supplementary Table 1, according to the results, when treated with 50 mmolL^–1^ NaCl, the relative reduction of each cultivars is all around 10% and this reduction is negligible, on the one hand, it shows that the concentration has little effect on the root length, on the other hand, because the relative reduction has little difference among 39 cultivars, it is impossible to accurately distinguish the tolerance, so the 50 mmolL^–1^ NaCl concentration was too low to distinguish the salt tolerance, but under 200 mmol L^–1^ NaCl treatment, most of the reductions are around 80%, this resulted in most of the cultivars cannot survive for enough time, so this treatment concentration was too high and unreasonable. 100 mmol L^–1^ NaCl can make a good distinction between salt tolerance and salt sensitive cultivars, and can also ensure that plants will not die during the test, thus, 100 mmol L^–1^ NaCl was selected for follow-up experiments.

**FIGURE 1:**
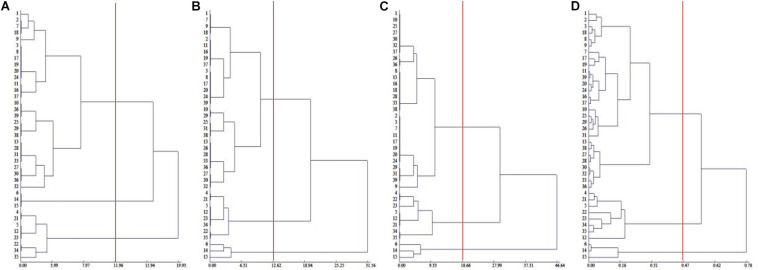
Cluster analysis of salt tolerance of Chinese cabbage. **(A)** Clustering of salt injury index in seedling stage. **(B)** Clustering of salt injury index in rosette stage. **(C)** Clustering of salt injury index during heading stage. **(D)** Relative value of fresh weight, electrolyte leakage relative value clustering in seedling stage.

To comprehensively evaluate the salt tolerance of Chinese cabbage, we measured the salt injury index of 39 Chinese cabbage cultivars at the seeding, rosette and heading stages (Supplementary Table 2), and through cluster analysis, in the seedling stage, rosette stage and the heading stage, 39 Chinese cabbage varieties could be divided into salt-tolerant, middle salt-tolerant and salt-sensitive types at Euclidean distances of 11.96, 12.62, and 18.66, respectively, and the clustering results at the three growth stages were identical (Figures 1A–C). The salt-tolerant type included cultivar number 6, 14, and 15, the salt-sensitive type included No. 4, 5, 12, 21, 22, 23, 34, and 35, and the rest were middle salt-tolerant cultivars. This indicated that the salt injury index can be used to reflect the salt tolerance of Chinese cabbage, we could evaluate salt tolerance at the seedling stage of the Chinese cabbage for easier and faster evaluation.

**FIGURE 2:**
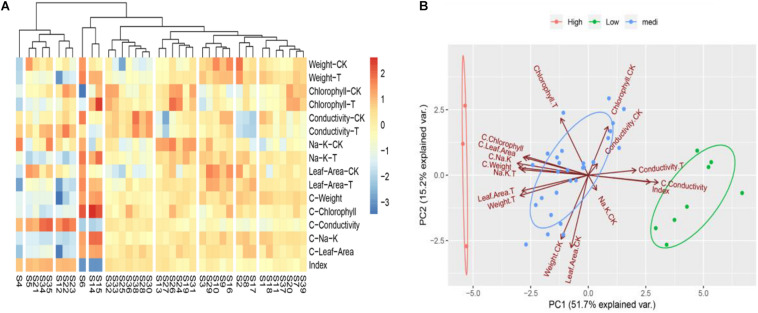
Heat map clustering and two-factor principal component analysis of all indicators in seedling stage. **(A)** Heat map clustering of all indicators in seedling stage. **(B)** Two-factor principal component analysis of all indicators in seedling stage. “CK,” “T,” and “C” are markers for different calculation methods, “CK” are measurement indicators of Chinese cabbage seedlings treated with 0 mol L^– 1^ NaCl, “T” are indicators of Chinese cabbage seedlings treated with 100 mol L^– 1^ NaCl, “C” is the relative value (the value of 100 mol L^– 1^/0 mol L^– 1^ NaCl treatment), and index is the salt injury index (%) of Chinese cabbage seedlings treated with NaCl stress.

To determine the convenient physiological indexes that can identify the salt tolerance of Chinese cabbage, we recorded the salt injury index, plant growth, chlorophyll content, electrolyte leakage, the content of K^+^ and Na^+^ and the ratio of K^+^: Na^+^ of the 39 Chinese cabbage cultivars under NaCl stress at the seeding stage (Supplementary Table 3). Heat map clustering and principal component analysis were performed using all these indicators (Figures 2A,B). The two results are consistent with the clustering results of Figures 1A–C, but the correlation analysis between classes and indicators was not clear, therefore, we further analyzed the correlation by comprehensive comparative analysis (Supplementary Table 4). The results showed that under salt stress, there were significant differences in the salt injury index of different Chinese cabbage cultivars. The salt injury index significantly increased with increasing of salt stress days, but the coefficient of variation among treatments decreased. Through stepwise regression analysis, a mathematical model was established for the salt injury index and related test indicators at the Chinese cabbage seedling stage.

Y=-18.899-13.388X11+ 38.483X13

(r= 0.9907,F= 956.71,p= 0.0000)

Although there were only two factors, the relative plant fresh weight and leaf electrolyte leakage were selected, and the coefficient of determination was as high as 0.9815 (*P* < 0.001). The salt tolerance results after the identification and evaluation of 39 Chinese cabbage cultivars with the selected two factors as statistical parameters were completely consistent with the salt injury index (Figure 1D), indicating that the relative values of the fresh weight and leaf electrolyte leakage in Chinese cabbage seedlings when treated with 100 mmol L^–1^ NaCl can be used as convenient indicators for salt tolerance identification. At the same time, by analyzing the growth conditions and phenotype, we selected the salt-tolerant cultivar Qinghua45 and the salt-sensitive cultivar Biyuchunhua for the follow-up test.

### Plant Growth Conditions and Osmotic Regulations Under Salt Stress

**FIGURE 3:**
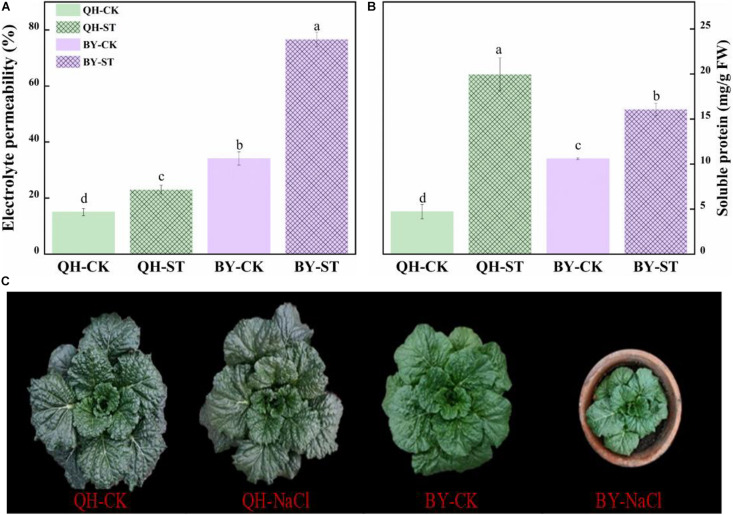
Effect of salt on electrolyte permeability, soluble protein and the growth of Qinghua45 (QH) and Biyuchunhua (BY). **(A)** Electrolyte permeability. **(B)** Soluble protein. **(C)** Growth condition of the whole plant after treatment with NaCl for 40 days. QH-CK: Qinghua45 treated with 0 mmol L^– 1^ NaCl, QH-ST: Qinghua45 treated with 100 mmol L^– 1^ NaCl, and so on. All data were determined 20 days after NaCl treatment. The data are the mean ± SD, and the different letters (a–d) indicate a significant difference at *P* < 0.05 according to Duncan’s test.

Salt stress affected the growth of different Chinese cabbages to different degrees. As shown in Table 1, under salt stress, the fresh weight, leaf area, and root activity of Qinghua45 decreased by 6.9, 19.81, and 13.87%, respectively, and chlorophyll increased by 18.92% compared with the control. The impact of salt stress on Biyuchunhua seemed more serious than that on Qinghua45, the fresh weight, leaf area, chlorophyll, and root activity of Biyuchunhua decreased by 66.74, 60.43, 59.63, and 56.5% compared with those of the control, respectively. Salt stress increased electrolyte permeability (Figure 3A), and Qinghua45 increased by 52.93%, but Biyuchunhua increased by 124.55% when compared with the control, the soluble protein increased 323.06% in Qinghua45 but only 51.6% was increased in Biyuchunhua (Figure 3B), indicating that Biyuchunhua was more injured than Qinghua45, the growth performance of the two cultivars under treatments were presented in Figure 3C.

**TABLE 1:** Effect of salt on the growth parameters of Qinghua45 (QH) and Biyuchunhua (BY).

Treatment	Fresh weight (g)	Leaf area (cm^2^)	Chlorophyll (mg/g)	Root activity μg/(g.h)
QH-CK	19.86 ± 0.265b	36.1 ± 5.987b	1.11 ± 0.082b	92.96 ± 3.67b
QH-ST	18.49 ± 1.33b	28.95 ± 0.61b	1.32 ± 0.031b	77.03 ± 3.59c
BY-CK	33.88 ± 1.518a	71.5 ± 2.622a	1.61 ± 0.144a	03.37 ± 4.09a
BY-ST	11.27 ± 1.011c	28.29 ± 3.517b	0.65 ± 0.101c	41.65 ± 3.35d

*QH-CK (Qinghua45 + 0 mmol L^–1^ NaCl) QH-ST (Qinghua45 + 100 mmol L^–1^ NaCl) BY-CK (Biyuchunhua + 0 mmol L^–1^ NaCl) BY-ST (Biyuchunhua + 100 mmol L^–1^ NaCl) All data were determined 20 days after NaCl treatment. The data are the mean ± SD and the different letters (a–d) indicate a significant difference at P < 0.05 according to Duncan’s test.*

### Effect of Salt Stress on Ion Homeostasis in Chinese Cabbage

**FIGURE 4:**
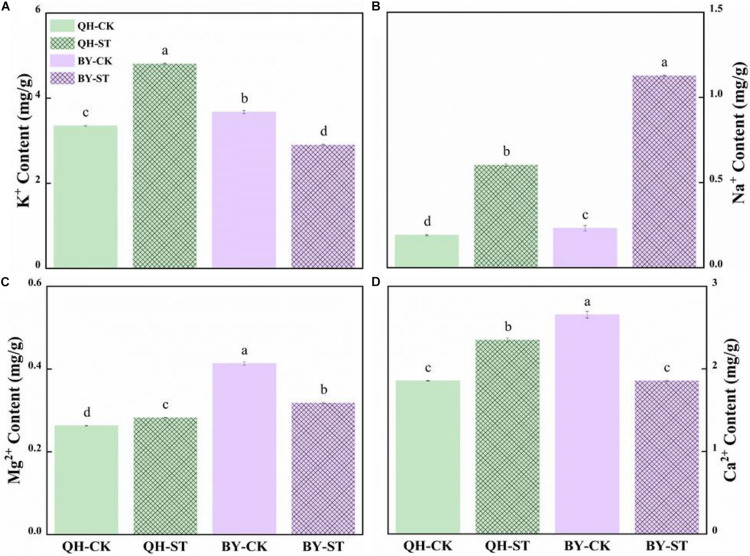
Effect of salt on the content of ions in the leaves of Qinghua45 (QH) and Biyuchunhua (BY). **(A)** Content of K^+^. **(B)** Content of Na^+^. **(C)** Content of Mg^2+^. **(D)** Content of Ca^2+^. All data were determined 20 days after NaCl treatment. Data are the means ± SD, and the different letters (a–d) indicate a significant difference at *P* < 0.05 according to Duncan’s test.

Salt stress seriously affected the absorption of mineral elements. The content of Na^+^ increased under salt stress, but increased by different percentages in the two cultivars. Na^+^ in Qinghua45 increased by 214.23% under salt stress compared with the control (Figure 4B), while that in Biyuchunhua increased by 386.04%, and the increasing rate was much higher than that of Qinghua45. Under salt stress, K^+^, Mg^2+^, and Ca^2+^ increased by 43.41, 7.34, and 26.68% in Qinghua45, and Biyuchunhua decreased by 21.01, 22.99, and 30.1% compared with the control, respectively (Figures 4A,C,D).

### Effect of Salt Stress on the Photosynthesis of Chinese Cabbage

**FIGURE 5:**
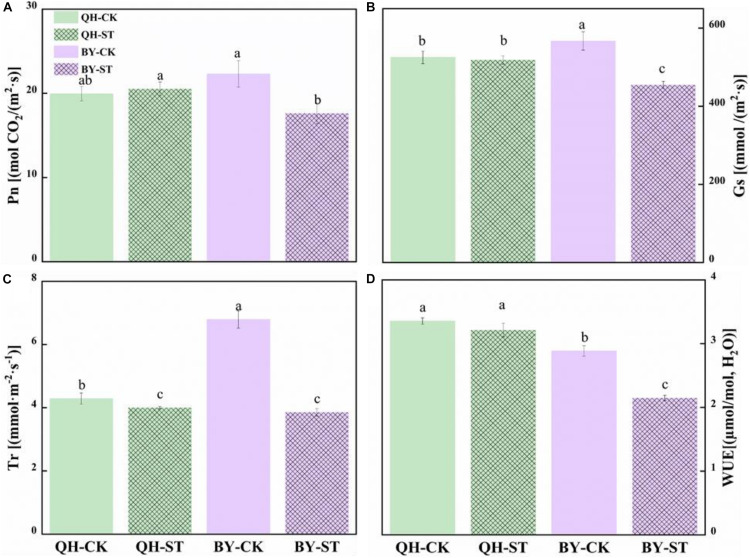
Effect of NaCl on the photosynthetic water vapor parameters of Qinghua45 (QH) and Biyuchunhua (BY). **(A)** Net photosynthetic rate (Pn), **(B)** stomatal conductance (Gs), **(C)** transpiration rate (Tr), and **(D)** water use efficiency (WUE) of Chinese cabbage under salt stress conditions. Data are the means of three replicates ± SD, and the different letters (a–d) indicate a significant difference at *P* ≤ 0.05 according to Duncan’s test.

The photosynthesis of Qinghua45 was not obviously injured by salt, but the photosynthetic rate of Biyuchunhua decreased by 21.02%. Stomatal conductance of Qinghua45 was affected but not obvious under salt stress, but a decrease of 19.88% appeared in Biyuchunhua. The trend of transpiration rate and instantaneous water use rate of the two cultivars under salt stress were consistent with the trend of stomatal conductance, and decreased by 6.71 and 4.23% compared with the control and showed no significant difference in Qinghua45; However, Biyuchunhua was severely affected, showing significant differences compared with the control with decreases of 43.23 and 25.55%, respectively (Figure 5).

### Identification of Differentially Expressed Genes by Transcriptome Sequencing

To investigate the mechanism and determine the key genes underlying salt stress tolerance in Chinese cabbage, dynamic profiling of the mRNA expression affected by salt stress was performed by transcriptome sequencing in the leaves of Qinghua45 and Biyuchunhua cultivars after 20 days’ treatment. Table 2 summarized the results of three biological repeats of RNA-seq analysis for each sample in each treatment. In our experiment, we obtained 596.3 million original readings from 12 samples. After filtering the adapter sequence and low-quality reads, we obtained 542.2 million clean reads. The percentage of readings obtained from the samples mapped to the reference genome was more than 92.02%, which met the needs of subsequent analysis.

**TABLE 2:** Sequencing data for 12 libraries obtained by RNA sequencing.

Sample name	Total reads	Clean reads	Clean base	GC (%)	Q20 (%)	Q30 (%)	Unique_Map
QHCK1	52,565,502	47,889,448	7,833,859,448	48.32	97.32	92.87	46,373,722 (88.22%)
QHCK2	48,630,636	44,337,691	7,268,557,386	48.31	97.22	92.67	42,955,000 (88.33%)
QHCK3	48,918,336	44,585,974	7,312,326,318	48.35	97.28	92.79	43,078,219 (88.06%)
QHS1	53,574,798	48,715,050	7,998,019,980	48.21	97.21	92.65	47,215,886 (88.13%)
QHS2	59,067,704	53,799,965	8,813,340,384	48.23	97.25	92.73	52,228,231 (88.42%)
QHS3	46,807,148	42,593,325	7,000,183,172	47.98	97.23	92.69	41,336,284 (88.31%)
BYCK1	46,669,894	42,446,112	6,979,059,194	48.28	97.23	92.7	41,073,199 (88.01%)
BYCK2	48,085,448	43,866,215	7,191,629,082	48.42	97.29	92.82	42,446,787 (88.27%)
BYCK3	56,496,268	51,426,737	8,443,548,790	48.43	97.27	92.78	49,753,439 (88.07%)
BYS1	48,709,928	44,321,968	7,269,555,322	48.15	97.39	93.03	42,936,941 (88.15%)
BYS2	42,579,918	38,686,912	6,365,353,998	48.04	97.36	92.94	37,478,881 (88.02%)
BYS3	44,225,200	39,551,609	6,612,655,366	48.18	97.13	92.45	37,850,581 (85.59%)

*QHCK and QHS represent the leaves of Qinghua45 under the control and 100 mmol L^–1^ treatments, respectively, and BYCK and BYS represent the leaves of Biyuchunhua under the control and 100 mmol L^–1^ treatments, respectively. Q20 and Q30 are the percentage of bases with a Clean Data quality value greater than 20 and 30 as a percentage of the total bases, respectively. GC (%) is the percentage of G and C bases in the total bases in Clean Data. Unique_map is the number of reads aligned to the unique position of the reference genome and its percentage. 1, 2, and 3 represent the three biological repeats from each sample per treatment.*

By comparing samples of the same Chinese cabbage cultivars under different conditions (control and salt) and different Chinese cabbage cultivars (Qinghua45 and Biyuchunhua) under the same conditions, we constructed four comparison groups: QHCK vs. QHS, QHCK vs. BYCK, QHS vs. BYS, and BYCK vs. BYS. In total, by restricting FDR < 0.01 and | log2(fold change)| ≥ 2, the analysis revealed 567 (227 upregulated genes and 340 downregulated genes), 1,157 (623 upregulated genes and 534 downregulated genes), 1,411 (758 upregulated genes and 653 downregulated genes) and 1,259 (495 upregulated genes and 764 downregulated genes) significant DEGs in QHCK vs. QHS, QHCK vs. BYCK, QHS vs. BYS, and BYCK vs. BYS, respectively (Supplementary Figure 2). The number of DEGs in QHCK vs. BYCK was lower than that in QHS vs. BYS, indicating that the number of DEGs in both cultivars was increased under salt stress. In addition, the number of DEGs in QHCK vs. QHS was much lower than that in BYCK vs. BYS, indicating that Biyuchunhua reacted more violently than Qinghua45 when exposed to salt stress, so the overall changes in genes of Biyuchunhua are larger, thus Biyuchunhua was more sensitive to salt stress than Qinghua45.

### Classification of DEGs

**FIGURE 6:**
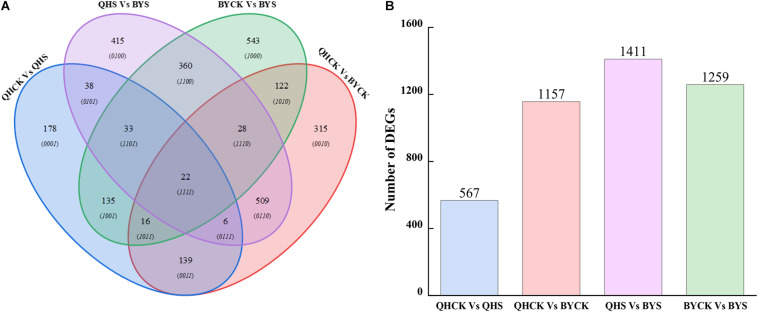
Venn diagrams for unique DEGs in four comparison groups. **(A)** Venn diagrams of DEGs in QHCK vs. QHS, QHCK vs. BYCK, QHS vs. BYS, and BYCK vs. BYS, **(B)** Total unique number of DEGs in group QHCK vs. QHS, QHCK vs. BYCK, QHS vs. BYS, and BYCK vs. BYS.

A total of 2,859 unique DEGs were identified in all four groups. These DEGs could be divided into 15 disjointed subgroups, among which 19.83% (567/2,859), 40.47% (1,157/2,859), 43.35% (1,411/2,859), and 44.04% (1,259/2,859) were group-specific DEGs in QHCK vs. QHS, QHCK vs. BYCK, QHS vs. BYS, and BYCK vs. BYS, respectively. The Venn diagram of the 2,859 unique DEGs in the four groups is shown in Figure 6. Twenty-two DEGs were commonly expressed across all four groups.

**FIGURE 7:**
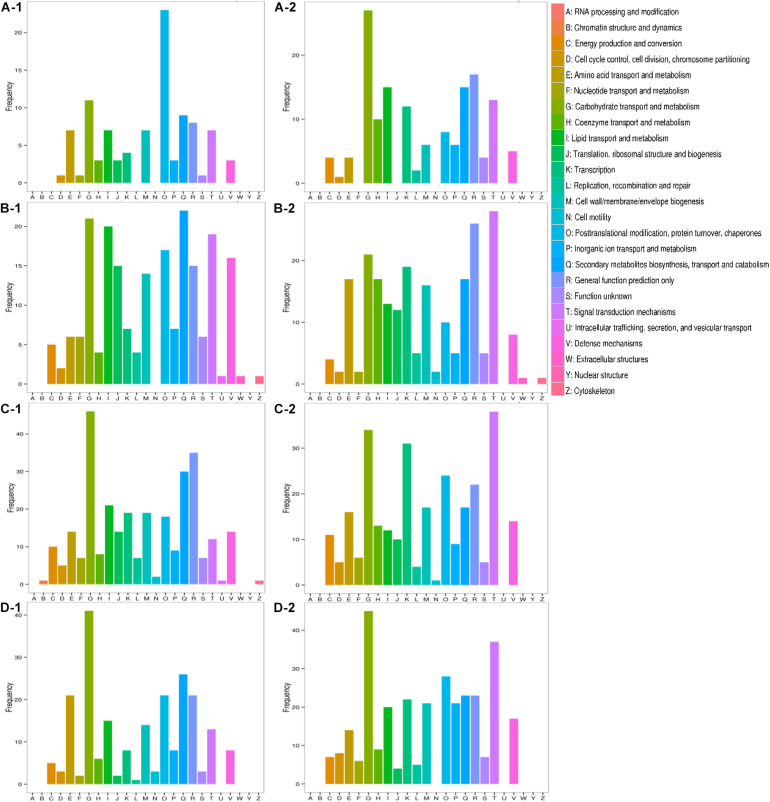
Functional classification in Clusters of Orthologous Groups of proteins (COG). Capital letters on the X-axis indicate the COG categories listed on the right of the histogram, and the Y-axis indicates the number of transcripts. **(A)** QHCK vs. QHS, **(B)** QHCK vs. BYCK, **(C)** QHS vs. BYS, and **(D)** BYCK vs. BYS, -1 up -2 down.

All transcripts were aligned to the COG database to predict possible functions. A total of 1,584 putative proteins were functionally classified into 25 groups. As shown in Figure 7, the difference between inorganic ion transport and metabolism (P), posttranslational modification, protein turnover, chaperones (O), transcription (K), and signal transduction mechanisms (T) was more obvious. In inorganic ion transport and metabolism (P), there were no significant changes in Qinghua45, but a large reduction appeared in Biyuchunhua. Qinghua45 increased more in posttranslational modification, protein turnover, and chaperones (O) under salt than Biyuchunhua. A larger decline appeared in transcription (K) in Biyuchunhua, and the signal transduction mechanisms (T) showed a serious reduction in Biyuchunhua.

**FIGURE 8:**
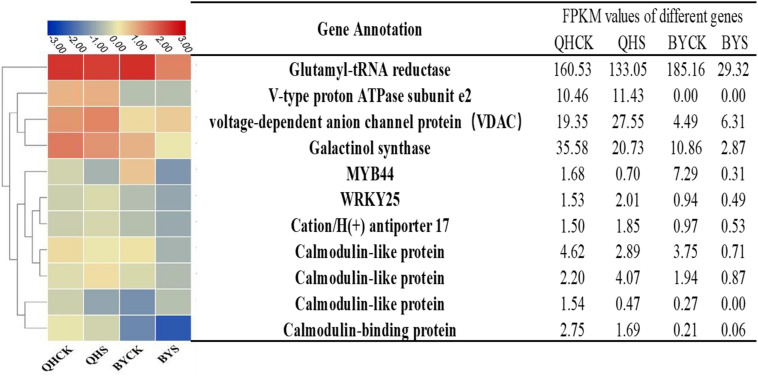
Heat map analysis of FPKM values of 11 key DEGs responding to salt stress in the transcriptome data in Qinghua45 (QH) and Biyuchunhua (BY). Color scale represents FPKM values. Data are the means of three replicates.

To study the molecular adaptation mechanism of the plants to salt stress, we further studied the expression and function of related genes that control the plant’s salt-related protein, chlorophyll synthesis, transcription factors and the signaling pathway, and eleven key and significantly different expressed genes in salt-tolerant and sensitive cultivars in these aspects were selected (Table 3). Based on the FPKM values of gene expression levels, we classified these genes and constructed a heat map for clustering (Figure 8). The gene expression of glutamyl-tRNA reductase (BraA01g027430.3C) showed the highest FPKM value, and V-type proton ATPase subunit e2 (Brassica_rapa_newGene_498) followed, and the reduction rate in Biyuchunhua was much larger than that of Qinghua45. The FPKM values of other genes in Biyuchunhua were all lower than that in Qinghua45 and were more seriously affected by salt stress.

**TABLE 3:** List of differentially expressed genes associated with interested pathways under salt stress.

Gene ID	Annotation	Fold change				Fold change			
		**QHCK vs. QHS**		**QHCK vs. BYCK**		**QHS vs. BYS**		**BYCK vs. BYS**	
			
**Signaling pathways**									
BraA03g042380.3C.gene	Calmodulin-like protein	–1.78	ns	–2.51	Down	–	–	–	–
BraA07g023900.3C.gene	Calmodulin-like protein	0.76	ns	–0.19	ns	–2.21	Down	–1.27	ns
BraA09g049350.3C.gene	Calmodulin-like protein	–0.79	ns	–0.30	ns	–2.02	Down	–2.52	Down
BraA02g040990.3C.gene	Calmodulin-binding protein	–0.83	ns	–3.64	Down	–4.68	Down	–	–
BraA03g010780.3C.gene	Voltage-dependent anion channel protein(VDAC)	0.39	ns	–2.11	Down	–2.1	Down	0.36	ns
**Stress-related proteins**									
BraA09g018570.3C.gene	Galactinol synthase	–0.90	ns	–1.71	ns	–2.85	Down	–2.04	Down
BraA01g014390.3C.gene	Cation/H(+) antiporter 17	0.19	ns	–2.09	Down	–5.16	Down	–	–
Brassica_rapa_newGene_498	V-type proton ATPase subunit e2	–0.10	ns	-Inf	Down	-Inf	Down	–0.16	ns
**Photosynthetic-related**									
BraA01g027430.3C.gene	Glutamyl-tRNA reductase	–0.39	ns	0.20	ns	–2.19	Down	–2.79	Down
**Transcription factors**									
BraA01g023290.3C.gene	MYB44	–1.38	ns	2.09	Up	–	–	–4.57	Down
BraA05g014020.3C.gene	WRKY25	0.27	ns	–0.72	ns	–2.02	Down	–1.05	ns

**FIGURE 9:**
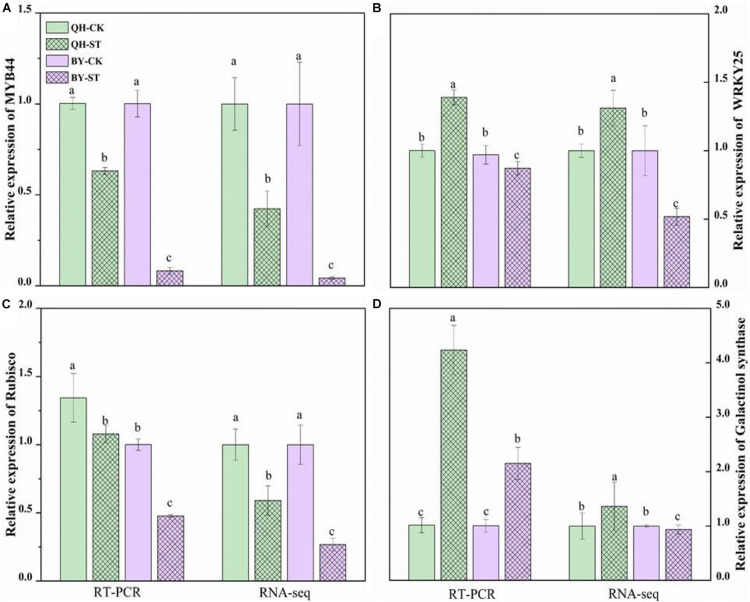
Relative expression of MYB44, WRKY25, Rubisco and galactinol synthase in the leaves of Qinghua45 (QH) and Biyuchunhua (BY). **(A)** MYB44, **(B)** WRKY25, **(C)** Rubisco, and **(D)** galactinol synthase of Chinese cabbage under salt stress conditions. Data are the means of three replicates ± SD, and the different letters (a–d) indicate a significant difference at *P* ≤ 0.05 according to Duncan’s test.

To verify the reliability of the RNA-seq data, quantitative real-time PCR (qRT-PCR) was performed on four randomly selected genes. The qRT-PCR results of these four genes are shown in Figure 9, and the primer design-related sequence is shown in Supplementary Table 5. The expression of the four selected genes showed differential expression between the two cultivars under salt stress. The relative trends in the expression patterns of the qRT-PCR results were roughly consistent with data trends in the transcriptome, which confirmed the reliability of our RNA-seq approach.

## Discussion

Salt stress significantly inhibits seed germination, seedling growth (Jamil et al., 2007; Wu et al., 2019), and biomass yield (Tseng et al., 2007) in Chinese cabbage. Decreased growth and biomass yield have been noted by other researchers in tomato and wheat (Jan et al., 2017; Ahmad et al., 2018). The increase in fresh weight was used as an indicator for salt stress tolerance in all plants (Munns, 2005), indicating that according to the fresh weight we can have a preliminary understanding of the growth status of Chinese cabbages. Kumar et al. (2005) ascribed the reduced growth of plant to the decreased water absorption due to osmotic effects, the deficiency of nutrients as a consequence of the ionic imbalance, and decreased activities in many metabolic. However, some studies have reported that there are great differences in the response ability of their protective mechanisms under stress due to different cultivars. Munns et al. (2006) found that different plant species evolved via different strategies to address the deleterious effects of excess salts.

In this experiment, through the sprouting bag test, we determined the 100 mmol L^–1^ NaCl was suitable treatment concentration. By combining linear regression analysis and multiple traits, we efficiently and accurately evaluated the salt tolerance of Chinese cabbage, which avoided the shortcomings of simple correlation analysis and truly reflected the correlation between the inclusion factor and the dependent variable. The determination model on salt tolerance of Chinese cabbage was established in this study, and only the relative fresh weight and electrolyte leakage of seedlings were selected, but the coefficient of the model determination was as high as 0.9815, which proved the feasible of this model. Further verification analysis showed that the results of cluster analysis with the selected two factors as statistical parameters were consistent with the results of cluster analysis of the salt injury index, indicating that the relative value of the fresh weight and the leaf electrolyte leakage rate of Chinese cabbage seedlings under salt stress could be used to judge their salt tolerance, and the two can be used as a convenient indicator for quickly identify of the salt tolerance in Chinese cabbages. Çoban and Göktürk Baydar (2016) and Oyiga et al. (2016) also reported the EL and fresh weight as a useful screening index for wheat and peppermint treated with salt.

According to the previous results, Qinghua45 and Biyuchunhua were selected for their different tolerance to salt stress. It was convenient to explore the mechanisms underlying salt tolerance by comparing the two chultivars. After treated with salt, the fresh weight, leaf area and chlorophyll content of Qinghua45 showed no significant difference compared with those of the control, but the activity of the root system of Qinghua45 was lower than that of the control; However, all these indicators of Biyuchunhua were significantly reduced. Generally, Biyuchunhua was far more seriously injured than Qinghua45. Oyiga et al. (2016) reported that wheat species differ greatly in salt tolerance because of their genetic makeup. We speculated that there may be some resistance mechanisms in Qinghua45 that promoted its high salt tolerance.

The cell membrane is one of the first targets of many stresses. Damage to the cell membrane can lead to electrolyte extravasation, and it have been reported that elevated electrolyte leakage results in oxidative damage that troubles the membrane system (Bai et al., 2006; Demidchik et al., 2014). Wheat increased the levels of electrolyte leakage in sensitive genotypes under salt, whereas it did not change in the tolerant genotypes (Oyiga et al., 2016). In this experiment, the electrolyte permeability increased under salt treatment, but the increase in Qinghua45 was significantly lower than that of Biyuchunhua, suggesting that the cell membrane of Qinghua45 was more tolerant. Li et al. (2014) declared that salt stress increased the total soluble protein content. Under salt stress, soluble proteins in salt-tolerant cultivars of barley (Sikand et al., 2013), sunflower (Salgado et al., 2011), and rice (Aghaee et al., 2011) crops increase and work as osmotic regulators to balance the osmotic pressure of cells, thus, cells can normally absorb water and transport nutrients from the underground. In our results, soluble protein significantly increased in Qinghua45, and showed no difference in Biyuchunhua, indicating that soluble protein ensured the osmotic pressure of cells and improved salt tolerance of Chinese cabbage. Studies also reported that the soluble protein content of lentil pairs decreases under salt stress, and there is no distinction between salt-tolerant and salt-sensitive cultivars (Hossain et al., 2017), this is inconsistent with our results, which may be caused by species.

Salt stress results in ion toxicity in plant cells via a large influx of Na^+^, which leads to intracellular ion imbalance and results in an imbalance between the internal and external osmotic pressure of the cell (Zhu, 2001). When the content of Na^+^ exceeds the threshold that the plant can withstand, the ion balance will be destroyed and disturb the stability of the cell membrane, photosynthesis, absorption and utilization of other elements (Bybordy, 2012; Singh et al., 2016). In plant cells, maintaining the content of K^+^ in the environment with a high Na^+^ concentration is a key factor in determining the tolerance to salinity. Salt-tolerant crops have been characterized by a higher uptake affinity of K^+^ over Na^+^ (Kausar et al., 2014). Once Na^+^ enters the cytoplasm of the cell, not the vacuole, it may interfere with the function of K^+^ as a series of enzyme cofactors are excessive. The lack of Mg^2+^, an important component of pigments, will seriously affect photosynthesis and reduce organic synthesis, thus affecting the growth of plants (Qiu et al., 2004). Ca^2+^ can reduce the Na^+^ content of the plant itself through different pathways, such as the SOS pathway and MAPK pathway, maintain the osmotic pressure and water potential of the cells, and reduce the damage to the plant from salt stress (Batelli et al., 2007). Ca^2+^ also plays an important role in endoplasmic reticulum stress signaling. In our studies, salt stress caused an increase in Na^+^ content, but the increase in Qinghua45 was less than that in Biyuchunhua. At the same time, the absorption of K^+^ in Qinghua45 increased but decreased in Biyuchunhua. Less absorption of Na^+^ was an important reason for the tolerance of Qinghua45. The content of Ca^2+^ and Mg^2+^ in Qinghua45 increased, and that were decreased significantly in Biyuchunhua under salt stress. Salt stress promoted the content of Ca^2+^, which might because it plays important roles as a signal, Ca^2+^ indirectly promoted the excretion of Na^+^ into vacuoles through the SOS_2_ pathway and reduced the injury of Na^+^; additionally, the increase in Mg^2+^ increased the plant’s photosynthetic capacity and promoted organic synthesis in Qinghua45.

It is well-known that photosynthesis is inhibited by salt stress (Khan et al., 2014). The two cultivars in our experiment had different responses to photosynthetic indicators, such as chlorophyll content, Pn, Gs, Tr, and WUE. The chlorophyll content of Qinghua45 increased under salt stress, and the content in Biyuchunhua decreased. Pn also showed this trend. Gs, Tr, and WUE decreased under salt stress, but Biyuchunhua decreased more than Qinghua45, indicating that Qinghua45 had a higher chlorophyll synthesis ability than Biyuchunhua. Chlorophyll plays a very important role in the absorption, transfer and transformation of light energy in the photosynthesis of plants (Dordas and Sioulas, 2008). High Na^+^ interferes with K^+^ and Ca^2+^, and disturbs efficient stomatal regulation (Slabu et al., 2009), affects the inhalation of carbon dioxide and decreases the carbon dioxide assimilation ability under salt stress, thus, the absorption of carbon dioxide is affected (Shaheen et al., 2013). A large number of studies have reported that salt stress can affect the transpiration rate by limiting stomatal conductance. To reduce water loss under saline conditions, plants also regulate leaf stomatal conductance to reduce water evaporation and avoid affecting photosynthesis (Chen et al., 2013). The decrease in Gs in Qinghua45 under salt stress was smaller than that in Biyuchunhua, and the same trend appeared in WUE, indicating that salt-tolerant cultivars had a high ability to regulate the balance of reducing water dissipation and increasing carbon dioxide uptake by controlling stomatal conductance to maintain a high efficiency of photosynthesis under salt stress. In general, the strong chlorophyll synthesis ability and ion absorption regulation ability of Qinghua45 are important factors for its strong salt tolerance.

When Chinese cabbage is subjected to salt, it first activates the signal transduction pathway and then activate a series of genes related to the defense response (Jamil et al., 2011). Voltage-dependent anion channels (VDACs) are mitochondrial outer membrane proteins that are not only the main pathway of calcium uptake but also a variety of molecular movement channels across the mitochondrial outer membrane, including NAD, ATP, and superoxide dismutase (Han et al., 2003). The endoplasmic reticulum (ER) is the main place for cells to process proteins and store the Ca^2+^, which plays an important role in maintaining cell survival and ensuring the normal physiological function of cells. It is very sensitive to stress, under stress stimulation, the endoplasmic reticulum can cause homeostasis imbalance in the ER. The voltage-dependent anion channel (VDAC) is involved in sensing the signal and passing it on to promote the release of Ca^2+^. Calmodulin (CaM) and calmodulin-like (CMLS) are the main receptors of Ca^2+^ and play a key role in deciphering encrypted calcium signals (Gevaudant et al., 2007; Zeng et al., 2015). After sensing the stress signal, the expression of calmodulin and calmodulin-like protein increased in the tolerant cultivars. On the one hand, they act as nutrients to promote plant growth; on the other hand, they transmit signals to SOS_2_ as a signal molecule. In rice, V-H^+^-ATPase can improve the tolerance of plants to salt stress (Wang et al., 2011; He et al., 2014; Wei et al., 2017), and enhanced V-H^+^-ATPase expression provides the necessary energy for transmembrane ion transport in vacuoles to promote the entry of Na^+^ into vacuoles. The cation/H (+) antiporter 17 (CHX) was related to Na^+^ compartmentation, which helps the exchange of H^+^ and Na^+^, thereby reducing Na^+^ injury, we found that in the sensitive cultivar it decreased significantly, but it would increase in the tolerant cultivar. Chlorophyll biosynthesis is closely related to L-glutamyl-tRNA, which promotes the synthesis of chlorophyll *a*, and then chlorophyll *a* is oxidized by chlorophyll in an oxygenase to form chlorophyll *b* (Beale, 2005; Nagata et al., 2005). In our study, salt-tolerant plants promoted the synthesis of more chlorophyll by upregulating chlorophyll-related genes, which in turn promoted plant organic matter synthesis and maintained the growth of plants under stress.

## Conclusion

**FIGURE 10:**
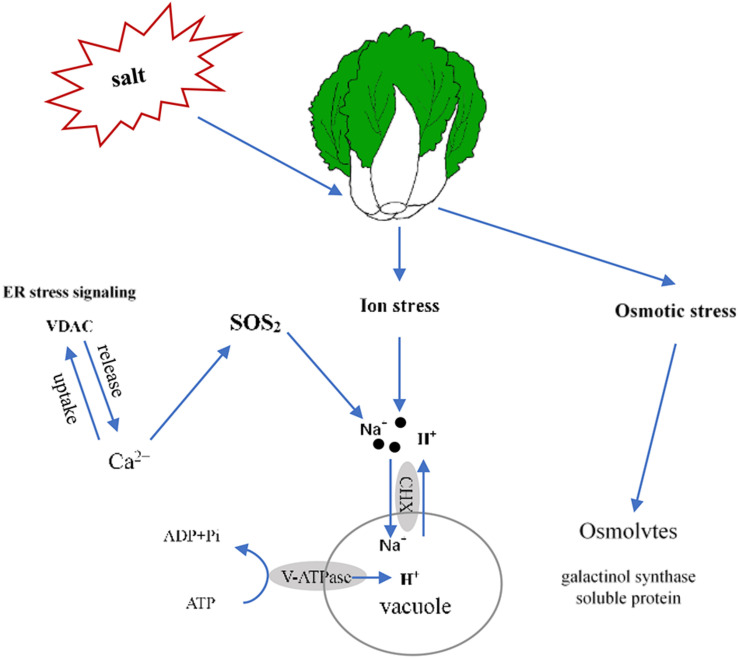
Proposed model of the salt stress response of Qinghua45 (QH) and Biyuchunhua (BY). Salt stress resulted in Chinese cabbage suffering from ion stress and osmotic stress. Na^+^ content increased under salt stress. Salt-tolerant plants promote the transport of sodium ions in vacuoles, increase calcium content through ER stress signaling VDAC and indirectly promote the transport of sodium ions to vacuoles through the SOS_2_ pathway. Galactinol synthase and soluble protein content increased to balance the osmotic pressure.

In this report, we determined that 100 mmol L^–1^ NaCl was a suitable salt stress concentration for Chinese cabbage through the sprouting bag test. We classified the salt tolerance of 39 cultivars and found that the relative value of fresh weight and leaf electrolyte leakage can be used as convenient indexes to evaluate salt tolerance in Chinese cabbage at the seedling stage, also selected Qinghua45 (salt-tolerant) and Biyuchunhua (salt-sensitive) as follow-up materials. Then, we compared and analyzed the physiological morphology and indicators of Qinghua45 and Biyuchunhua cultivars under salt stress and found that the absorption and transport of ions play great roles in the salt tolerance of Chinese cabbages. Next, according to an analysis of the transcriptomic dataset, we successfully identified some key DEGs related to ion absorption under salt stress. This study helps elucidate the salt tolerance mechanism of Chinese cabbage. We briefly summarized the proposed model (Figure 10). Salt stress led to an increase in the absorption of Na^+^ and resulted in ion toxicity in Chinese cabbage. After sensing salt stress signaling, VDAC promoted the release of Ca^2+^, which indirectly promoted the transport of Na^+^ to vacuoles through the SOS_2_ pathway. Cation/H (+) antiporter 17 and V-H + -ATPase promoted the exchange of Na^+^ and H^+^ and kept Na^+^ in vacuoles, thus reducing the injury of salt stress. The increases in galactinol synthase and soluble protein synthesis also helped relieve osmotic stress caused by salt.

## Data Availability Statement

The original contributions presented in the study are included in the article/Supplementary Material. The sequencing data are publicly available at the NCBI SRA database under accession number PRJNA552162, further inquiries can be directed to the corresponding author.

## Author Contributions

NL: conceptualization, methodology, software, formal analysis, investigation, data curation, writing original draft, and visualization. ZZ, BC, and ZC: supervision and project administration. KX: conceptualization, resources, writing review and editing, supervision, project administration, and funding acquisition. KX and BC modified the article. All authors contributed to the article and approved the submitted version.

## Conflict of Interest

The authors declare that the research was conducted in the absence of any commercial or financial relationships that could be construed as a potential conflict of interest.

## Footnotes

1 http://www.genome.jp/kegg/

## References

[B1] Aghaee A. Moradi F. Zare-Maivan H. Zarinkamar F. Pour Irandoost H. Sharifi P. (2011). Physiological responses of two rice (*Oryza sativa* L.) genotypes to chilling stress at seedling stage.pdf. *Afr. J. Biotechnol.* 10 7617–7621. 10.5897/AJB11.069

[B2] Ahmad P. Abd Allah E. F. Alyemeni M. N. Wijaya L. Alam P. Bhardwaj R. (2018). Exogenous application of calcium to 24-epibrassinosteroid pre-treated tomato seedlings mitigates NaCl toxicity by modifying ascorbate-glutathione cycle and secondary metabolites. *Sci. Rep.* 8:13515. 10.1038/s41598-018-31917-1 30201952 PMC6131545

[B3] Bai L.-P. Sui F.-G. Ge T.-D. Sun Z.-H. Lu Y.-Y. Zhou G.-S. (2006). Effect of soil drought stress on leaf water status, membrane permeability and enzymatic antioxidant system of maize. *Pedosphere* 16 326–332. 10.1016/s1002-0160(06)60059-3

[B4] Bartels D. Sunkar R. (2005). Drought and salt tolerance in plants. *Crit. Rev. Plant Sci.* 24 23–58. 10.1080/07352680590910410

[B5] Batelli G. Verslues P. E. Agius F. Qiu Q. Fujii H. Pan S. (2007). SOS2 promotes salt tolerance in part by interacting with the vacuolar H+-ATPase and upregulating its transport activity. *Mol. Cell Biol.* 27 7781–7790. 10.1128/MCB.00430-07 17875927 PMC2169139

[B6] Bayuelo-Jiménez J. S. Debouck D. G. Lynch J. P. (2003). Growth, gas exchange, water relations, and ion composition of Phaseolus species grown under saline conditions. *Field Crops Res.* 80 207–222. 10.1016/S0378-4290(02)00179-X

[B7] Beale S. I. (2005). Green genes gleaned. *Trends Plant Sci.* 10 309–312. 10.1016/j.tplants.2005.05.005 15951223

[B8] Bybordy A. (2012). Study effect of salinity on some physiologic and morphologic properties of two grape cultivars. *Life Sci. J.* 9 1092–1101.

[B9] Camara A. K. S. Zhou Y. Wen P. C. Tajkhorshid E. Kwok W. M. (2017). Mitochondrial VDAC1: a key gatekeeper as potential therapeutic target. *Front. Physiol.* 8:460. 10.3389/fphys.2017.00460 28713289 PMC5491678

[B10] Chen P. Yan K. Shao H. Zhao S. (2013). Physiological mechanisms for high salt tolerance in wild soybean (Glycine soja) from Yellow River Delta, China: photosynthesis, osmotic regulation, ion flux and antioxidant capacity. *PLoS One* 8:e83227. 10.1371/journal.pone.0083227 24349468 PMC3861505

[B11] Çoban Ö Göktürk Baydar N. (2016). Brassinosteroid effects on some physical and biochemical properties and secondary metabolite accumulation in peppermint (*Mentha piperita* L.) under salt stress. *Ind. Crops Prod.* 86 251–258. 10.1016/j.indcrop.2016.03.049

[B12] Deinlein U. Stephan A. B. Horie T. Luo W. Xu G. Schroeder J. I. (2014). Plant salt-tolerance mechanisms. *Trends Plant Sci.* 19 371–379. 10.1016/j.tplants.2014.02.001 24630845 PMC4041829

[B13] Demidchik V. Straltsova D. Medvedev S. S. Pozhvanov G. A. Sokolik A. Yurin V. (2014). Stress-induced electrolyte leakage: the role of K+-permeable channels and involvement in programmed cell death and metabolic adjustment. *J. Exp. Bot.* 65 1259–1270. 10.1093/jxb/eru004 24520019

[B14] Dordas C. A. Sioulas C. (2008). Safflower yield, chlorophyll content, photosynthesis, and water use efficiency response to nitrogen fertilization under rainfed conditions. *Ind. Crops Prod.* 27 75–85. 10.1016/j.indcrop.2007.07.020

[B15] Flowers T. J. Munns R. Colmer T. D. (2015). Sodium chloride toxicity and the cellular basis of salt tolerance in halophytes. *Ann. Bot.* 115 419–431. 10.1093/aob/mcu217 25466549 PMC4332607

[B16] Geng G. Li R. Stevanato P. Lv C. Lu Z. Yu L. (2020). Physiological and transcriptome analysis of sugar beet reveals different mechanisms of response to neutral salt and alkaline salt stresses. *Front. Plant Sci.* 11:571864. 10.3389/fpls.2020.571864 33193507 PMC7604294

[B17] Gevaudant F. Duby G. von Stedingk E. Zhao R. Morsomme P. Boutry M. (2007). Expression of a constitutively activated plasma membrane H+-ATPase alters plant development and increases salt tolerance. *Plant Physiol.* 144 1763–1776. 10.1104/pp.107.103762 17600134 PMC1949876

[B18] Goslings D. Meskauskiene R. Kim C. Lee K. P. Nater M. Apel K. (2004). Concurrent interactions of heme and FLU with Glu tRNA reductase (HEMA1), the target of metabolic feedback inhibition of tetrapyrrole biosynthesis, in dark- and light-grown Arabidopsis plants. *Plant J.* 40 957–967. 10.1111/j.1365-313X.2004.02262.x 15584960

[B19] Han D. Antunes F. Canali R. Rettori D. Cadenas E. (2003). Voltage-dependent anion channels control the release of the superoxide anion from mitochondria to cytosol. *J. Biol. Chem.* 278 5557–5563. 10.1074/jbc.M210269200 12482755

[B20] Hasanuzzaman M. Nahar K. Alam M. M. Bhowmik P. C. Hossain M. A. Rahman M. M. (2014). Potential use of halophytes to remediate saline soils. *Biomed. Res. Int.* 2014:589341. 10.1155/2014/589341 25110683 PMC4109415

[B21] Hasanuzzaman M. Nahar K. Fujita M. Ahmad P. Chandna R. Prasad M. N. V. (2013). “Enhancing plant productivity under salt stress: relevance of poly-omics,” in *Salt Stress in Plants*, eds Ahmad P. Azooz M. M. Prasad M. N. V. (New York, NY: Springer), 113–156.

[B22] He X. Huang X. Shen Y. Huang Z. (2014). Wheat V-H+-ATPase subunit genes significantly affect salt tolerance in Arabidopsis thaliana. *PLoS One* 9:e86982. 10.1371/journal.pone.0086982 24498005 PMC3907383

[B23] Hossain M. S. Alam M. U. Rahman A. Hasanuzzaman M. Nahar K. Al Mahmud J. (2017). Use of iso-osmotic solution to understand salt stress responses in lentil (Lens culinaris Medik.). *S. Afr. J. Bot.* 113 346–354. 10.1016/j.sajb.2017.09.007

[B24] Jamil A. Riaz S. Ashraf M. Foolad M. R. (2011). Gene expression profiling of plants under salt stress. *Crit. Rev. Plant Sci.* 30 435–458. 10.1080/07352689.2011.605739

[B25] Jamil M. Lee K. B. Jung K. Y. Lee D. B. Han M. S. Rha E. S. (2007). Salt stress inhibits germination and early seedling growth in cabbage (Brassica oleracea capitata L.). *Pak. J. Biol. Sci.* 10 910–914. 19069887 10.3923/pjbs.2007.910.914

[B26] Jan A. U. Hadi F. Midrarullah, Nawaz M. A. Rahman K. (2017). Potassium and zinc increase tolerance to salt stress in wheat (*Triticum aestivum* L.). *Plant Physiol. Biochem.* 116 139–149. 10.1016/j.plaphy.2017.05.008 28558283

[B27] Kanehisa M. Araki M. Goto S. Hattori M. Hirakawa M. Itoh M. (2008). KEGG for linking genomes to life and the environment. *Nucleic Acids Res.* 36, D480–D484. 10.1093/nar/gkm882 18077471 PMC2238879

[B28] Kausar A. Ashraf M. Y. Niaz M. (2014). Some physiological and genetic determinants of salt tolerance in sorghum (*Sorghum bicolor* (L.) Moench): biomass production and nitrogen metabolism. *Pak. J. Bot.* 46 515–519.

[B29] Khan M. I. R. Asgher M. Khan N. A. (2014). Alleviation of salt-induced photosynthesis and growth inhibition by salicylic acid involves glycinebetaine and ethylene in mungbean (*Vigna radiata* L.). *Plant Physiol. Biochem.* 80 67–74. 10.1016/j.plaphy.2014.03.026 24727790

[B30] Kumar R. Goyal V. Kuhad M. S. (2005). Influence of frtility-salinity interactions on growth, water status and yield of indian mustard (*Brassica juncea*).pdf. *Indian J. Plant Physiol.* 10 139–144.

[B31] Li T. Hu Y. Du X. Tang H. Shen C. Wu J. (2014). Salicylic acid alleviates the adverse effects of salt stress in *Torreya grandis* cv. Merrillii seedlings by activating photosynthesis and enhancing antioxidant systems. *PLoS One* 9:e109492. 10.1371/journal.pone.0109492 25302987 PMC4193794

[B32] Liu W. Zhou Q. An J. Sun Y. Liu R. (2010). Variations in cadmium accumulation among Chinese cabbage cultivars and screening for Cd-safe cultivars. *J. Hazard. Mater.* 173 737–743. 10.1016/j.jhazmat.2009.08.147 19775811

[B33] Livak K. J. Schmittgen T. D. (2001). Analysis of relative gene expression data using real-time quantitative PCR and the 2(-Delta Delta C(T)) Method. *Methods* 25 402–408. 10.1006/meth.2001.1262 11846609

[B34] Mao X. Cai T. Olyarchuk J. G. Wei L. (2005). Automated genome annotation and pathway identification using the KEGG Orthology (KO) as a controlled vocabulary. *Bioinformatics* 21, 3787–3793. 10.1093/bioinformatics/bti430 15817693

[B35] Munns R. (2005). Genes and salt tolerance: bringing them together. *New Phytol.* 167 645–663. 10.1111/j.1469-8137.2005.01487.x 16101905

[B36] Munns R. James R. A. Lauchli A. (2006). Approaches to increasing the salt tolerance of wheat and other cereals. *J. Exp. Bot.* 57 1025–1043. 10.1093/jxb/erj100 16510517

[B37] Munns R. Tester M. (2008). Mechanisms of salinity tolerance. *Annu. Rev. Plant Biol.* 59 651–681. 10.1146/annurev.arplant.59.032607.092911 18444910

[B38] Nagata N. Tanaka R. Satoh S. Tanaka A. (2005). Identification of a vinyl reductase gene for chlorophyll synthesis in Arabidopsis thaliana and implications for the evolution of Prochlorococcus species. *Plant Cell* 17 233–240. 10.1105/tpc.104.027276 15632054 PMC544501

[B39] Noble A. (2018). The effect of ripe plantain peels waste on the phytoextraction of Pb and Cd by *Echinochloa colona* (L.) link. *Int. J. Nat. Resour. Ecol. Manag.* 3 19–13. 10.11648/j.ijnrem.20180301.13

[B40] Oyiga B. C. Sharma R. C. Shen J. Baum M. Ogbonnaya F. C. Léon J. (2016). Identification and characterization of salt tolerance of wheat germplasm using a multivariable screening approach. *J. Agron. Crop Sci.* 202 472–485. 10.1111/jac.12178

[B41] Parvaiz A. Satyawati S. (2008). Salt stress and phyto-biochemical responses of plants-a review. *Plant Soil Environ.* 54 89–100.

[B42] Qiu Q. S. Guo Y. Quintero F. J. Pardo J. M. Schumaker K. S. Zhu J. K. (2004). Regulation of vacuolar Na+/H+ exchange in Arabidopsis thaliana by the salt-overly-sensitive (SOS) pathway. *J. Biol. Chem.* 279 207–215. 10.1074/jbc.M307982200 14570921

[B43] Salgado P. R. Molina Ortiz S. E. Petruccelli S. Mauri A. N. (2011). Sunflower protein concentrates and isolates prepared from oil cakes have high water solubility and antioxidant capacity. *J. Am. Oil Chem. Soc.* 88 351–360. 10.1007/s11746-010-1673-z

[B44] Shaheen S. Naseer S. Ashraf M. Akram N. A. (2013). Salt stress affects water relations, photosynthesis, and oxidative defense mechanisms inSolanum melongenaL. *J. Plant Interact.* 8 85–96. 10.1080/17429145.2012.718376

[B45] Sikand V. Tong P. S. Walker J. (2013). Effect of adding salt during the diafiltration step of milk protein concentrate powder manufacture on mineral and soluble protein composition. *Dairy Sci. Technol.* 93 401–413. 10.1007/s13594-013-0110-0

[B46] Singh D. Yadav N. S. Tiwari V. Agarwal P. K. Jha B. (2016). A SNARE-like superfamily protein SbSLSP from the halophyte salicornia brachiata confers salt and drought tolerance by maintaining membrane stability, K(+)/Na(+) ratio, and antioxidant machinery. *Front. Plant Sci.* 7:737. 10.3389/fpls.2016.00737 27313584 PMC4889606

[B47] Slabu C. Zörb C. Steffens D. Schubert S. (2009). Is salt stress of faba bean (*Vicia faba*) caused by Na+ or Cl– toxicity? *J. Plant Nutr. Soil Sci.* 172 644–651. 10.1002/jpln.200900052

[B48] Tseng M. J. Liu C.-W. Yiu J.-C. (2007). Enhanced tolerance to sulfur dioxide and salt stress of transgenic Chinese cabbage plants expressing both superoxide dismutase and catalase in chloroplasts. *Plant Physiol. Biochem.* 45 822–833. 10.1016/j.plaphy.2007.07.011 17851086

[B49] Wang L. He X. Zhao Y. Shen Y. Huang Z. (2011). Wheat vacuolar H+-ATPase subunit B cloning and its involvement in salt tolerance. *Planta* 234 1–7. 10.1007/s00425-011-1383-2 21344312

[B50] Wei D. Zhang W. Wang C. Meng Q. Li G. Chen T. H. H. (2017). Genetic engineering of the biosynthesis of glycinebetaine leads to alleviate salt-induced potassium efflux and enhances salt tolerance in tomato plants. *Plant Sci.* 257 74–83. 10.1016/j.plantsci.2017.01.012 28224920

[B51] Wu G.-Q. Jiao Q. Shui Q.-Z. (2016). Effect of salinity on seed germination, seedling growth, and inorganic and organic solutes accumulation in sunflower (*Helianthus annuus* L.). *Plant Soil Environ.* 61 220–226. 10.17221/22/2015-pse

[B52] Wu H. Guo J. Wang C. Li K. Zhang X. Yang Z. (2019). An effective screening method and a reliable screening trait for salt tolerance of brassica napus at the germination stage. *Front. Plant Sci.* 10:530. 10.3389/fpls.2019.00530 31105727 PMC6499083

[B53] Xiong L. Zhu J.-K. (2002). Molecular and genetic aspects of plant responses to osmotic stress.pdf. *Plant Cell Environ.* 25 131–139. 11841658 10.1046/j.1365-3040.2002.00782.x

[B54] Young M. D. Wakefield M. J. Smyth G. K. Oshlack A. (2010). Gene ontology analysis for RNA-seq. *Genome Biol*. 11, 1–12. 10.1186/gb-2010-11-2-r14 20132535 PMC2872874

[B55] Zeng H. Xu L. Singh A. Wang H. Du L. Poovaiah B. W. (2015). Involvement of calmodulin and calmodulin-like proteins in plant responses to abiotic stresses. *Front. Plant Sci.* 6:600. 10.3389/fpls.2015.00600 26322054 PMC4532166

[B56] Zhang Z. Cao B. Li N. Chen Z. Xu K. (2019). Comparative transcriptome analysis of the regulation of ABA signaling genes in different rootstock grafted tomato seedlings under drought stress. *Environ. Exp. Bot.* 166:103814. 10.1016/j.envexpbot.2019.103814

[B57] Zhao Q. Zhao Y. J. Zhao B. C. Ge R. C. Li M. Shen Y. Z. (2009). Cloning and functional analysis of wheat V-H+-ATPase subunit genes. *Plant Mol. Biol.* 69 33–46. 10.1007/s11103-008-9403-8 18836689

[B58] Zhu J. Bie Z. Li Y. (2008). Physiological and growth responses of two different salt-sensitive cucumber cultivars to NaCl stress. *Soil Sci. Plant Nutr.* 54 400–407. 10.1111/j.1747-0765.2008.00245.x

[B59] Zhu J.-K. (2001). Plant salt tolerance.pdf. *Trends Plant Sci.* 6 66–71. 11173290 10.1016/s1360-1385(00)01838-0

