# Supplementary material for: Comparative Transcriptome Analysis of Two Contrasting Chinese Cabbage (Brassica rapa L.) Genotypes Reveals That Ion Homeostasis Is a Crucial Biological Pathway Involved in the Rapid Adaptive Response to Salt Stress
Source: Front Plant Sci. 2021 Jun 14;12:683891. doi: 10.3389/fpls.2021.683891 (PMC8236865; doi:10.3389/fpls.2021.683891)
Supplement: Supplementary file 1 [file Data_Sheet_1.docx]

**Supplementary data**

**Table S1. The actual and relative values of root length under NaCl treatments in Sprouting bag test.**

| Material code | 0 NaCl | 50 mol L^-1^ NaCl | 100 mol L^-1^ NaCl | 200 mol L^-1^ NaCl | 50 mol L^-1^ /0 NaCl | 100 mol L^-1^ /0 NaCl | 200 mol L^-1^ /0 NaCl |
| --- | --- | --- | --- | --- | --- | --- | --- |
| 1 | 86.29±4.74 | 80.06±4.4 | 38.8±2.13 | 14.13±0.78 | 92.78% | 44.96% | 16.37% |
| 2 | 67.39±3.7 | 60.79±3.34 | 25.8±1.42 | 15.86±0.87 | 90.21% | 38.29% | 23.54% |
| 3 | 63.1±3.46 | 58.74±3.22 | 25.25±1.39 | 15.76±0.86 | 93.08% | 40.01% | 24.97% |
| 4 | 77.63±4.26 | 71.32±3.91 | 19.5±1.07 | 10.19±0.56 | 91.87% | 25.11% | 13.12% |
| 5 | 33.95±1.86 | 32.9±1.81 | 9.39±0.52 | 5.25±0.29 | 96.91% | 27.65% | 15.47% |
| 6 | 55.51±3.05 | 54.18±2.97 | 44.1±2.42 | 21.25±1.17 | 97.59% | 79.45% | 38.29% |
| 7 | 60.26±3.31 | 58.05±3.19 | 28.35±1.56 | 11.36±0.62 | 96.33% | 47.05% | 18.84% |
| 8 | 57.57±3.16 | 52.81±2.9 | 30.69±1.68 | 12.3±0.67 | 91.73% | 53.31% | 21.36% |
| 9 | 56.79±3.12 | 54.49±2.99 | 31.83±1.75 | 15.83±0.87 | 95.94% | 56.05% | 27.87% |
| 10 | 55.96±3.07 | 50.91±2.79 | 27.44±1.51 | 13.22±0.73 | 90.97% | 49.04% | 23.62% |
| 11 | 63.73±3.5 | 60.41±3.32 | 32.67±1.79 | 16.38±0.9 | 94.79% | 51.26% | 25.71% |
| 12 | 65.97±3.62 | 58.48±3.21 | 18.87±1.04 | 11.29±0.62 | 88.64% | 28.60% | 17.12% |
| 13 | 51.82±2.84 | 45.32±2.49 | 26.28±1.44 | 13.04±0.72 | 87.45% | 50.72% | 25.17% |
| 14 | 62.01±3.4 | 59.14±3.25 | 48.19±2.65 | 23.7±1.3 | 95.37% | 77.71% | 38.22% |
| 15 | 70.55±3.87 | 66.11±3.63 | 62.68±3.44 | 32.37±1.78 | 93.69% | 88.84% | 45.88% |
| 16 | 62.19±3.41 | 55.61±3.05 | 32.88±1.8 | 16.58±0.91 | 89.41% | 52.87% | 26.66% |
| 17 | 78.5±4.31 | 69.71±3.83 | 36.62±2.01 | 21.61±1.19 | 88.80% | 46.65% | 27.53% |
| 18 | 69.59±3.82 | 60.88±3.34 | 37.09±2.04 | 13.97±0.77 | 87.48% | 53.30% | 20.07% |
| 19 | 61.43±3.37 | 57.14±3.14 | 28.75±1.58 | 13.69±0.75 | 93.01% | 46.80% | 22.29% |
| 20 | 63.98±3.51 | 58.98±3.24 | 23.71±1.3 | 11.39±0.62 | 92.18% | 37.06% | 17.80% |
| 21 | 82.39±4.52 | 74.17±4.07 | 24.01±1.32 | 12.34±0.68 | 90.02% | 29.14% | 14.98% |
| 22 | 71.24±3.91 | 68.91±3.78 | 21.62±1.19 | 12.4±0.68 | 96.73% | 30.35% | 17.41% |
| 23 | 41.57±2.28 | 38.47±2.11 | 11.31±0.62 | 6.76±0.37 | 92.54% | 27.22% | 16.27% |
| 24 | 64.62±3.55 | 56.74±3.11 | 26.18±1.44 | 13.67±0.75 | 87.80% | 40.51% | 21.16% |
| 25 | 44.12±2.42 | 39.81±2.19 | 18.52±1.02 | 8.45±0.46 | 90.24% | 41.98% | 19.16% |
| 26 | 47.88±2.63 | 46.41±2.55 | 21.51±1.18 | 6.3±0.35 | 96.93% | 44.93% | 13.15% |
| 27 | 59.93±3.29 | 52.47±2.88 | 27.77±1.52 | 8.21±0.45 | 87.55% | 46.34% | 13.69% |
| 28 | 32.26±1.77 | 29.74±1.63 | 16.05±0.88 | 8.27±0.45 | 92.19% | 49.75% | 25.64% |
| 29 | 57.06±3.13 | 49.52±2.72 | 25.75±1.41 | 8.19±0.45 | 86.78% | 45.12% | 14.35% |
| 30 | 38.27±2.1 | 34.35±1.89 | 18.52±1.02 | 10.45±0.57 | 89.76% | 48.40% | 27.30% |
| 31 | 28.93±1.59 | 26.72±1.47 | 15.03±0.83 | 7.15±0.39 | 92.37% | 51.94% | 24.70% |
| 32 | 47.83±2.63 | 43.63±2.39 | 21.22±1.16 | 11.46±0.63 | 91.21% | 44.36% | 23.96% |
| 33 | 51.44±2.82 | 48.31±2.65 | 26.01±1.43 | 7.94±0.44 | 93.92% | 50.57% | 15.44% |
| 34 | 66.58±3.65 | 61.43±3.37 | 20.4±1.12 | 11.63±0.64 | 92.27% | 30.65% | 17.46% |
| 35 | 75.43±4.14 | 68.92±3.78 | 21.24±1.17 | 10.74±0.59 | 91.38% | 28.16% | 14.24% |
| 36 | 49.64±2.72 | 44.51±2.44 | 22.78±1.25 | 10.24±0.56 | 89.67% | 45.90% | 20.64% |
| 37 | 46.14±2.53 | 43.95±2.41 | 21.58±1.19 | 7.68±0.42 | 95.26% | 46.78% | 16.64% |
| 38 | 60.28±3.31 | 56.31±3.09 | 27.43±1.51 | 12.83±0.7 | 93.41% | 45.50% | 21.28% |
| 39 | 57.91±3.18 | 51.88±2.85 | 27.81±1.53 | 13.06±0.72 | 89.58% | 48.01% | 22.55% |

**Table S2. The index of salt damage of different genotype Chinese cabbage at different growth stages under NaCl stress.**

| Material code | Seedling stage | Rosette stage | Heading stage | Variety name |  | Material code | Seedling stage | Rosette stage | Heading stage | Variety name |
| --- | --- | --- | --- | --- | --- | --- | --- | --- | --- | --- |
| 1 | 12.50 | 25.00 | 54.17 | Baihe |  | 22 | 38.46 | 55.56 | 79.17 | Jinyuhuang |
| 2 | 13.46 | 30.56 | 41.67 | Baiheng |  | 23 | 36.54 | 52.78 | 75.00 | Degao 45 |
| 3 | 15.38 | 27.78 | 41.67 | Shuanggai 3 |  | 24 | 15.38 | 27.78 | 41.67 | Qinghua 60 |
| 4 | 34.62 | 50.00 | 79.17 | Degao 3 |  | 25 | 21.15 | 36.11 | 54.17 | Degaoxiaobao |
| 5 | 36.54 | 52.78 | 83.33 | Degao 18 |  | 26 | 19.23 | 38.89 | 58.33 | Zaoshu 181 |
| 6 | 1.92 | 8.33 | 12.50 | Degao 58 |  | 27 | 25.00 | 41.67 | 54.17 | Hongjiangjun |
| 7 | 13.46 | 25.00 | 41.67 | Degao 127 |  | 28 | 23.08 | 38.89 | 50.00 | Jinwawa |
| 8 | 15.38 | 27.78 | 50.00 | Degao 129 |  | 29 | 21.15 | 33.33 | 45.83 | Biyuchunhua |
| 9 | 11.54 | 25.00 | 37.50 | Degao 157 |  | 30 | 25.00 | 41.67 | 54.17 | Lubai 1 |
| 10 | 19.23 | 33.33 | 54.17 | Yongchun |  | 31 | 23.08 | 36.11 | 45.83 | Qinghua 76 |
| 11 | 17.31 | 30.56 | 41.67 | CR jindi |  | 32 | 26.92 | 41.67 | 54.17 | Degao 7 |
| 12 | 36.54 | 52.78 | 83.33 | Jinqiu |  | 33 | 23.08 | 38.89 | 50.00 | Huangguan |
| 13 | 23.08 | 38.89 | 50.00 | Jinfenghuang |  | 34 | 38.46 | 52.78 | 70.83 | Chunqiu 70 |
| 14 | 1.92 | 5.56 | 8.33 | Chaoluxianfeng |  | 35 | 40.38 | 55.56 | 70.83 | Degao 1 |
| 15 | 1.92 | 2.78 | 4.17 | Qinghua 45 |  | 36 | 25.00 | 38.89 | 58.33 | Degao 8 |
| 16 | 17.31 | 30.56 | 50.00 | Dajiangjun |  | 37 | 17.31 | 30.56 | 54.17 | Deyang 01 |
| 17 | 15.38 | 27.78 | 41.67 | Xiabai 1 |  | 38 | 21.15 | 36.11 | 50.00 | CR95 |
| 18 | 13.46 | 25.00 | 50.00 | Xiaotaiyang |  | 39 | 19.23 | 27.78 | 45.83 | CR118 |
| 19 | 15.38 | 30.56 | 41.67 | Qinghua 80 |  | Avg. | 21.18 | 34.69 | 51.39 |  |
| 20 | 15.38 | 27.78 | 41.67 | CR117 |  | SD | 10.06 | 12.76 | 18.29 |  |
| 21 | 34.62 | 50.00 | 83.33 | Tiejia 65 |  | CV% | 47.49 | 36.83 | 35.6 |  |

| **Table S3. The actual values and relative values of Chinese cabbage related indicators under NaCl stress at seedling stage.** | | | | | | | | | | | | | | | |
| --- | --- | --- | --- | --- | --- | --- | --- | --- | --- | --- | --- | --- | --- | --- | --- |
| Material code | Plant fresh weight（g） | | | Chlorophyll（mg/g） | | | Electrolyte leakage（%） | | | K^+^/Na^+^（%） | | | Leaf area（cm^2^） | | |
|  | 0 NaCl | 100mol L^-1^ NaCl | Relative value | 0 NaCl | 100mol L^-1^ NaCl | Relative value | 0 NaCl | 100mol L^-1^ NaCl | Relative value | 0 NaCl | 100mol L^-1^  NaCl | Relative value | 0 NaCl | 100mol L^-1^ NaCl | Relative value |
| 1 | 11.69 | 10.43 | 0.89 | 1.53 | 1.32 | 0.86 | 28.92 | 31.86 | 1.10 | 152.37 | 78.55 | 0.52 | 82.63 | 39.86 | 0.48 |
| 2 | 18.63 | 16.74 | 0.90 | 1.41 | 1.32 | 0.94 | 18.17 | 20.69 | 1.14 | 195.53 | 98.27 | 0.50 | 113.64 | 48.92 | 0.43 |
| 3 | 11.92 | 10.13 | 0.85 | 1.42 | 1.15 | 0.81 | 24.47 | 28.07 | 1.15 | 207.57 | 100.50 | 0.48 | 85.53 | 42.10 | 0.49 |
| 4 | 4.85 | 1.00 | 0.21 | 1.42 | 1.10 | 0.78 | 25.38 | 35.62 | 1.40 | 250.90 | 54.28 | 0.22 | 41.31 | 4.13 | 0.10 |
| 5 | 15.96 | 3.26 | 0.20 | 1.59 | 1.21 | 0.76 | 24.01 | 34.64 | 1.44 | 158.64 | 36.08 | 0.23 | 116.65 | 21.68 | 0.19 |
| 6 | 16.06 | 19.28 | 1.20 | 1.08 | 1.19 | 1.10 | 32.74 | 31.32 | 0.96 | 233.73 | 142.77 | 0.61 | 111.79 | 115.61 | 1.03 |
| 7 | 12.26 | 9.73 | 0.79 | 1.83 | 1.74 | 0.95 | 24.20 | 27.80 | 1.15 | 184.98 | 85.57 | 0.46 | 68.09 | 42.44 | 0.62 |
| 8 | 9.77 | 8.07 | 0.83 | 1.75 | 1.56 | 0.89 | 17.69 | 19.66 | 1.11 | 197.02 | 102.78 | 0.52 | 70.31 | 59.87 | 0.85 |
| 9 | 13.60 | 11.27 | 0.83 | 1.60 | 1.44 | 0.90 | 29.00 | 32.59 | 1.12 | 196.80 | 102.63 | 0.52 | 94.59 | 58.02 | 0.61 |
| 10 | 17.16 | 10.45 | 0.61 | 1.69 | 1.53 | 0.90 | 24.02 | 29.10 | 1.21 | 195.81 | 83.86 | 0.43 | 117.74 | 82.99 | 0.70 |
| 11 | 9.39 | 6.56 | 0.70 | 1.55 | 1.39 | 0.89 | 21.90 | 26.29 | 1.20 | 185.52 | 77.99 | 0.42 | 70.47 | 37.47 | 0.53 |
| 12 | 8.40 | 0.41 | 0.05 | 1.69 | 1.29 | 0.76 | 24.47 | 36.41 | 1.49 | 213.85 | 43.83 | 0.20 | 79.90 | 3.93 | 0.05 |
| 13 | 8.42 | 3.97 | 0.47 | 1.70 | 1.48 | 0.87 | 19.48 | 24.55 | 1.26 | 234.45 | 92.22 | 0.39 | 65.03 | 38.70 | 0.60 |
| 14 | 10.50 | 12.70 | 1.21 | 1.48 | 1.71 | 1.16 | 22.75 | 21.90 | 0.96 | 148.90 | 111.16 | 0.75 | 65.00 | 71.84 | 1.11 |
| 15 | 9.08 | 12.23 | 1.35 | 1.88 | 2.08 | 1.11 | 23.22 | 22.90 | 0.99 | 196.80 | 147.30 | 0.75 | 59.93 | 69.25 | 1.16 |
| 16 | 16.98 | 11.27 | 0.66 | 1.38 | 1.36 | 0.99 | 29.13 | 34.98 | 1.20 | 207.30 | 90.27 | 0.44 | 120.16 | 73.15 | 0.61 |
| 17 | 11.85 | 9.01 | 0.76 | 1.51 | 1.39 | 0.92 | 16.71 | 19.37 | 1.16 | 159.13 | 71.93 | 0.45 | 92.25 | 77.73 | 0.84 |
| 18 | 10.21 | 8.65 | 0.85 | 1.57 | 1.37 | 0.87 | 27.28 | 30.86 | 1.13 | 171.66 | 85.92 | 0.50 | 46.61 | 39.50 | 0.85 |
| 19 | 8.27 | 6.05 | 0.73 | 1.59 | 1.44 | 0.90 | 25.10 | 29.19 | 1.16 | 238.13 | 112.11 | 0.47 | 72.65 | 47.89 | 0.66 |
| 20 | 8.67 | 6.26 | 0.72 | 1.95 | 1.72 | 0.88 | 20.85 | 24.76 | 1.19 | 191.77 | 87.76 | 0.46 | 83.27 | 43.09 | 0.52 |
| 21 | 11.67 | 2.70 | 0.23 | 1.58 | 1.27 | 0.80 | 20.39 | 28.79 | 1.41 | 189.80 | 43.07 | 0.23 | 108.18 | 20.54 | 0.19 |
| 22 | 10.52 | 1.27 | 0.12 | 1.98 | 1.56 | 0.79 | 27.44 | 43.56 | 1.59 | 180.00 | 34.92 | 0.19 | 66.18 | 9.74 | 0.15 |
| 23 | 7.30 | 1.46 | 0.20 | 1.87 | 1.47 | 0.78 | 23.75 | 36.39 | 1.53 | 202.10 | 42.76 | 0.21 | 53.63 | 8.42 | 0.16 |
| 24 | 7.76 | 5.52 | 0.71 | 1.97 | 1.83 | 0.93 | 23.93 | 28.55 | 1.19 | 212.56 | 94.10 | 0.44 | 49.16 | 38.90 | 0.79 |
| 25 | 3.78 | 2.21 | 0.59 | 1.63 | 1.47 | 0.90 | 25.29 | 31.13 | 1.23 | 159.13 | 65.70 | 0.41 | 37.93 | 21.38 | 0.56 |
| 26 | 11.18 | 6.38 | 0.57 | 1.95 | 1.92 | 0.98 | 21.14 | 26.10 | 1.23 | 249.92 | 102.63 | 0.41 | 98.16 | 44.91 | 0.46 |
| 27 | 8.48 | 3.89 | 0.46 | 1.75 | 1.55 | 0.88 | 15.11 | 19.09 | 1.26 | 236.97 | 85.57 | 0.36 | 86.10 | 50.29 | 0.58 |
| 28 | 5.94 | 2.68 | 0.45 | 1.68 | 1.48 | 0.88 | 28.63 | 36.32 | 1.27 | 165.75 | 57.12 | 0.34 | 45.12 | 34.79 | 0.77 |
| 29 | 12.84 | 7.66 | 0.60 | 1.51 | 1.27 | 0.85 | 25.24 | 30.96 | 1.23 | 231.88 | 97.83 | 0.42 | 126.88 | 88.75 | 0.70 |
| 30 | 6.73 | 2.77 | 0.41 | 1.61 | 1.42 | 0.88 | 30.93 | 40.13 | 1.30 | 180.00 | 59.97 | 0.33 | 57.45 | 35.12 | 0.61 |
| 31 | 11.05 | 6.09 | 0.55 | 1.93 | 1.65 | 0.86 | 25.85 | 32.28 | 1.25 | 239.93 | 92.65 | 0.39 | 74.47 | 43.31 | 0.58 |
| 32 | 9.91 | 4.06 | 0.41 | 1.95 | 1.74 | 0.89 | 26.82 | 34.95 | 1.30 | 208.28 | 70.52 | 0.34 | 49.76 | 25.38 | 0.51 |
| 33 | 6.20 | 2.69 | 0.43 | 1.94 | 1.64 | 0.85 | 27.89 | 35.76 | 1.28 | 201.24 | 71.93 | 0.36 | 42.88 | 18.77 | 0.44 |
| 34 | 9.80 | 2.27 | 0.23 | 1.63 | 1.20 | 0.74 | 23.79 | 37.44 | 1.57 | 194.19 | 40.16 | 0.21 | 73.38 | 16.85 | 0.23 |
| 35 | 12.05 | 2.83 | 0.23 | 1.75 | 1.31 | 0.75 | 24.01 | 38.34 | 1.60 | 250.54 | 51.47 | 0.21 | 95.58 | 23.64 | 0.25 |
| 36 | 8.48 | 3.62 | 0.43 | 1.50 | 1.50 | 1.00 | 26.80 | 34.63 | 1.29 | 166.02 | 56.93 | 0.34 | 67.00 | 48.44 | 0.72 |
| 37 | 8.47 | 5.55 | 0.66 | 1.66 | 1.45 | 0.87 | 25.31 | 30.45 | 1.20 | 161.95 | 70.30 | 0.43 | 78.20 | 33.83 | 0.43 |
| 38 | 6.52 | 3.15 | 0.48 | 1.50 | 1.39 | 0.93 | 33.44 | 42.06 | 1.26 | 187.15 | 72.69 | 0.39 | 45.54 | 25.42 | 0.56 |
| 39 | 12.22 | 8.56 | 0.70 | 1.84 | 1.62 | 0.88 | 21.69 | 25.94 | 1.20 | 180.11 | 78.16 | 0.43 | 76.00 | 34.95 | 0.46 |
| Avg. | 10.37 | 6.53 | 0.61 | 1.66 | 1.48 | 0.89 | 24.54 | 30.65 | 1.25 | 197.91 | 79.34 | 0.40 | 76.64 | 42.09 | 0.55 |
| SD | 3.41 | 4.44 | 0.31 | 0.20 | 0.22 | 0.09 | 4.086 | 6.277 | 0.16 | 29.17 | 26.64 | 0.13 | 24.61 | 24.40 | 0.27 |
| CV% | 32.86 | 67.96 | 50.44 | 12.20 | 14.61 | 10.58 | 16.65 | 20.48 | 12.64 | 14.74 | 33.58 | 33.10 | 32.11 | 57.97 | 48.03 |

**Table S4. Correlation coefficients of related indicators of Chinese cabbage under different NaCl concentrations in seedling stage.**

| Index | X_1_ | X_2_ | X_3_ | X_4_ | X_5_ | X_6_ | X_7_ | X_8_ | X_9_ | X_10_ | X_11_ | X_12_ | X_13_ | X_14_ | X_15_ |
| --- | --- | --- | --- | --- | --- | --- | --- | --- | --- | --- | --- | --- | --- | --- | --- |
| X_2_ | 0.683^**^ |  |  |  |  |  |  |  |  |  |  |  |  |  |  |
| X_3_ | -0.329^*^ | -0.442^**^ |  |  |  |  |  |  |  |  |  |  |  |  |  |
| X_4_ | -0.189 | 0.073 | 0.728^**^ |  |  |  |  |  |  |  |  |  |  |  |  |
| X_5_ | -0.089 | -0.006 | -0.250 | -0.192 |  |  |  |  |  |  |  |  |  |  |  |
| X_6_ | -0.221 | -0.520^**^ | 0.020 | -0.317^*^ | 0.775^**^ |  |  |  |  |  |  |  |  |  |  |
| X_7_ | 0.032 | -0.057 | 0.101 | -0.017 | -0.106 | 0.000 |  |  |  |  |  |  |  |  |  |
| X_8_ | 0.266 | 0.786^**^ | -0.173 | 0.380^*^ | -0.039 | -0.583^**^ | 0.256 |  |  |  |  |  |  |  |  |
| X_9_ | 0.851^**^ | 0.466^**^ | -0.355^*^ | -0.305 | -0.210 | -0.237 | 0.180 | 0.172 |  |  |  |  |  |  |  |
| X_10_ | 0.539^**^ | 0.804^**^ | -0.417^**^ | 0.110 | -0.021 | -0.491^**^ | 0.057 | 0.762^**^ | 0.521^**^ |  |  |  |  |  |  |
| X_11_ | 0.290 | 0.869^**^ | -0.274 | 0.322^*^ | -0.023 | -0.611^**^ | -0.172 | 0.884^**^ | 0.105 | 0.733^**^ |  |  |  |  |  |
| X_12_ | 0.168 | 0.673^**^ | -0.223 | 0.497^**^ | 0.085 | -0.430^**^ | -0.140 | 0.750^**^ | 0.022 | 0.699^**^ | 0.789^**^ |  |  |  |  |
| X_13_ | -0.210 | -0.796^**^ | 0.298 | -0.313 | -0.003 | 0.626^**^ | 0.171 | -0.866^**^ | -0.070 | -0.732^**^ | -0.941^**^ | -0.798^**^ |  |  |  |
| X_14_ | 0.230 | 0.815^**^ | -0.224 | 0.395^*^ | -0.019 | -0.608^**^ | -0.182 | 0.890^**^ | 0.064 | 0.723^**^ | 0.981^**^ | 0.829^**^ | -0.951^**^ |  |  |
| X_15_ | 0.084 | 0.623^**^ | -0.186 | 0.419^**^ | 0.044 | -0.483^**^ | -0.162 | 0.774^**^ | -0.052 | 0.784^**^ | 0.824^**^ | 0.829^**^ | -0.834^**^ | 0.852^**^ |  |
| Y | -0.222 | -0.824^**^ | 0.258 | -0.360^*^ | -0.039 | .585^**^ | 0.182 | -0.881^**^ | -0.060 | -0.735^**^ | -0.969^**^ | -0.824^**^ | 0.981^**^ | -0.972^**^ | -0.842^**^ |

* p<0.05, ** p<0.01. X_1_, X_3_, X_5_, X_7_ and X_9_ are the fresh weight (g), chlorophyll (mg/g), electrolyte leakage (%), potassium-sodium ratio (%), leaf area(cm^2^) of Chinese cabbage seedlings treated with 0 mol L^-1^ NaCl, respectively; X_2_, X_4_, X_6_, X_8_ and X_10_ are the fresh weight (g), chlorophyll (mg/g), electrolyte leakage (%), potassium-sodium ratio (%), leaf area(cm^2^) of Chinese cabbage seedlings treated with 100mol L^-1^ NaCl, respectively; X_11_, X_12_, X_13_, X_14_, X_15_ are the value of 100mol L^-1^/0 mol L^-1^ of the corresponding indicator, respectively; Y is the salt injury index (%) of Chinese cabbage seedlings treated with NaCl stress.

**Table S5. Primer design related sequence**

| **Gene name** | **Primer name** | **Primer sequence** |
| --- | --- | --- |
| BrActin | BrActin-F | CTCAGTCCAAAAGAGGTATTCT |
|  | BrActin-R | GTAGAATGTGTGATGCCAGATC |
| MYB44 | MYB44-F | AAGAAGAAGACGAGCAGCTAAG |
|  | MYB44-R | ACCGTAGCCGACAAGATTTAC |
| WRKY25 | WRKY25-F | CCAAACGCTTCTTCTGCTTTAC |
|  | WRKY25-R | CCTGGTGATCTGATCCAAACTC |
| Rubisco | Rubisco-F | CCTCCAGTTGGCAAGAAGAA |
|  | Rubisco-R | ACTTGTTGCGGATAAGGTAGTC |
| galactinol synthase | galactinol synthase -F | TCACCGGACCAGTCGCTAACG |
|  | galactinol synthase -R | AGCAAGACCCACCATTCCTTTCAC |


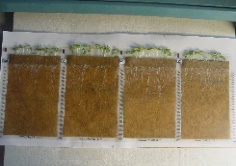

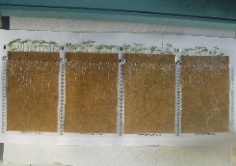

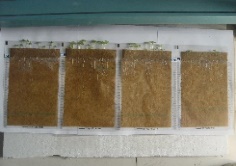

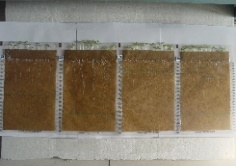

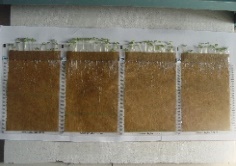

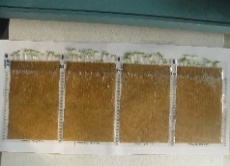


10


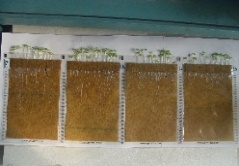


9


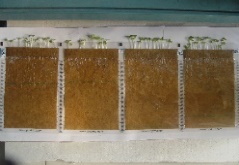


8


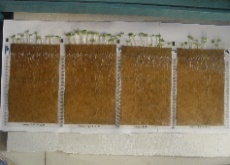


7


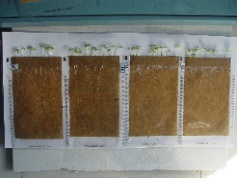


1


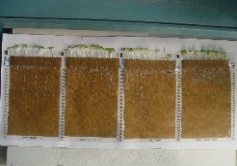


6


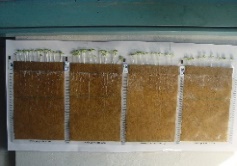


2


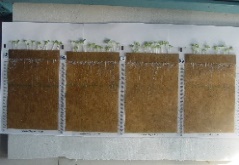


3


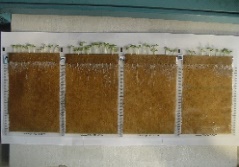


4


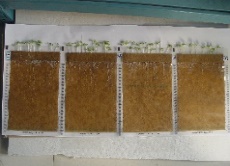


5


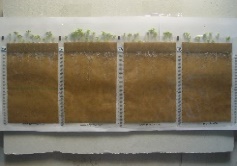


20


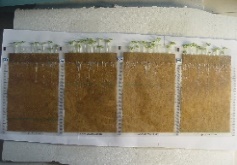


19


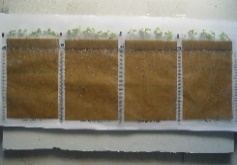


18


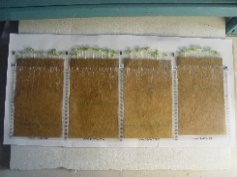


17


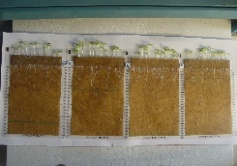


16


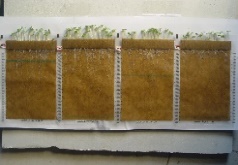


39


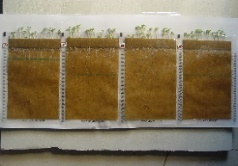


38


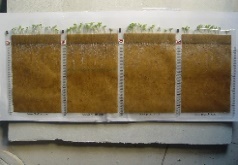


37


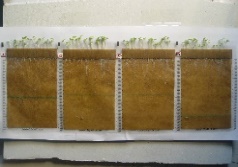


36


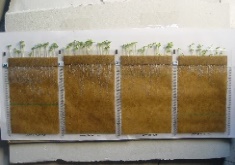


31


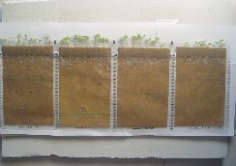


26


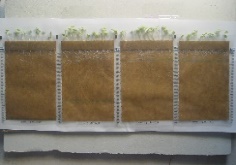


27


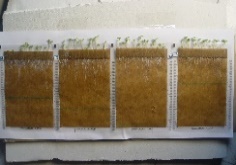


32


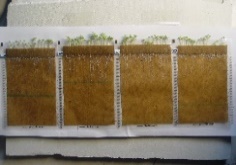


33


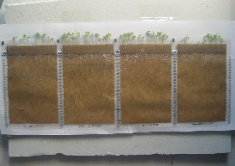


28


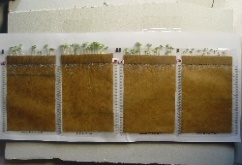


34


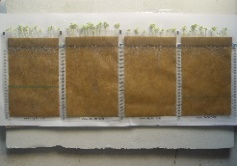


29


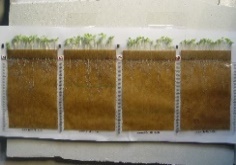


35


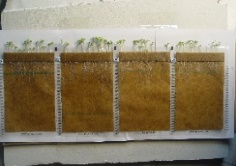


30


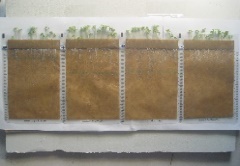


24


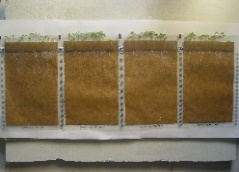


23


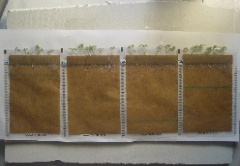


22


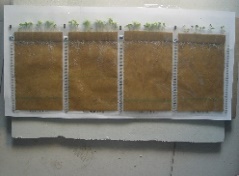


21


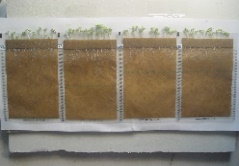


25

15

14

13

12

11

**Fig. S1. 39 Chinese cabbage varieties treated with salt in Sprouting bag** Each variety has four same salt treatment concentrations, and the salt treatment concentrations in the four sprouting bags of each variety are 0 50 100 200mmol L^-1^ NaCl from left to right.

**Fig. S2.** Volcano plots for expressed genes in the four comparison groups. Volcano plots for all the expressed genes in (A) QHCK vs. QHS, (B) QHCK vs. BYCK, (C) QHS vs. BYS, and (D) BYCK vs. BYS. The X- and Y-axes present the log2 (FC) for the two samples and −log10 (FDR), respectively. Red (upregulated) and green (downregulated) dots indicate that the genes have significant differences, while the blue dots correspond to genes with no significant differences.

Note:

**QHCK vs. QHS**: fold change in the expression level in QH under NaCl treatment compared with that in QH under CK treatment;

**QHCK vs. BYCK**: under control conditions, fold change in the expression level in BY compared with that in QH;

**QHS vs. BYS**: under NaCl conditions, fold change in the expression level in BY compared with that in QH;

**BYCK vs. BYS**: fold change in the expression level in BY under NaCl treatment compared with that in BY under CK treatment.
